# Dynamic shift of internal electric field accelerates enzymatic polyethylene terephthalate depolymerization

**DOI:** 10.1038/s42004-026-01888-w

**Published:** 2026-01-12

**Authors:** Mingna Zheng, Jinfeng Chen, Weiliang Dong, Ren Wei, Jinyue Chen, Xiaowen Tang, Qingzhu Zhang, Qiao Wang, Wenxing Wang, Guoqiang Wang, Yanwei Li

**Affiliations:** 1 Academician Workstation for Big Data in Ecology and Environment, Environment Research Institute, Shandong University, Qingdao, PR China; 2 Key Laboratory of Structural Biology of Zhejiang Province, School of Life Sciences, Westlake University, Zhejiang, PR China; 3 College of Biotechnology and Pharmaceutical Engineering, Nanjing Tech University, Nanjing, PR China; 4 Institute of Biochemistry, Department of Biotechnology & Enzyme Catalysis, University of Greifswald, Greifswald, Germany; 5 Department of Medical Chemistry, School of Pharmacy, Qingdao University, Qingdao, PR China

**Keywords:** Computational chemistry, Biocatalysis, Catalytic mechanisms

## Abstract

Enzymatic recycling of polyethylene terephthalate (PET) has been recognized as an eco-friendly option for addressing the global plastic waste problem. Fully deciphering the catalytic mechanism is vital for designing high-performance enzymes. Here, we performed quantum mechanics/molecular mechanics molecular dynamics simulations to systematically explore the depolymerization mechanism of PET by the hydrolase LCC^ICCG^. We demonstrate that both PET chain binding and product release require free energy barriers, whereas the rate-determining step corresponds to a catalytic process with a free energy barrier of 20.4 kcal·mol^-1^. We also observe that the enzyme internal electric field varies dynamically throughout the catalytic process. Oriented external electric field analysis indicates that this “dynamic shift” stabilizes the transition state more than the reactant, thereby lowering the energy barrier. We anticipate that these insights will contribute to the rational engineering of PET hydrolases by optimizing their dynamic internal electric fields.

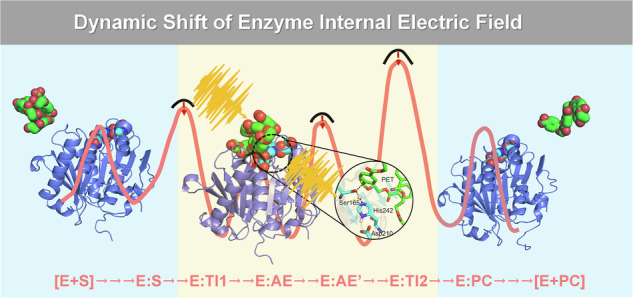

## Abstract (web summary)

Enzymes offer a sustainable solution for recycling polyethylene terephthalate (PET) waste, however, their efficiency remains limited for large-scale applications. Here, the authors use QM/MM MD simulations to reveal the depolymerization mechanism of a cutinase variant, uncovering dynamic internal electric fields that stabilize transition states and lower the energy barrier, offering a strategy to engineer high-performance PET hydrolases.

## Introduction

Enzymes are intriguing catalysts that play a central role in life processes. Recently, there has been increasing interest in harnessing enzymes to tackle major challenges faced by humankind^1–3^. For instance, improper end-of-life management of waste plastics has caused severe damage to the global ecosystem and poses a significant threat to human health^4–7^. Hence, in recent years, enzymes have been identified and engineered to tackle the global plastic waste dilemma by offering an alternative to chemical recycling methods, particularly for hydrolyzable waste plastics such as polyethylene terephthalate (PET)^8–10^. PET is the most widely produced polyester in modern society. Notably, a natural hydrolase from *Thermobifida fusca* (TfH) was discovered to depolymerize PET two decades ago^11^. Many other hydrolases capable of depolymerizing PET into its monomers, terephthalic acid (TPA) and ethylene glycol (EG), have been identified so far^12–17^. However, the activity and thermostability of these natural PET hydrolases are generally insufficient for industrial recycling applications at elevated temperature, thereby underscoring the need for enzyme optimization through diverse protein engineering techniques^9,18^.

With the advent of resolved crystal structures^17,19–22^, extensive efforts have been devoted to the engineering of PET hydrolases, such as computational redesign^23–25^, rational engineering^17,26,27^, directed evolution^28,29^, and machine learning^30^. One of the most representative examples is the structurally guided redesign of the leaf-branch-compost cutinase (LCC), which resulted in the superior LCC^ICCG^ variant with a quadruple mutation (F244I/D238C/S283C/Y127G)^27^. It can depolymerize 90% of amorphized waste PET within 10 h at 72 °C, and the obtained monomers can be purified and used to resynthesize virgin PET. Nonetheless, the search for novel PET hydrolases that exhibit superior catalytic efficiency relative to LCC^ICCG^ while allowing for operation at lower temperatures or with alternative pH profiles is ongoing to facilitate more environmentally sustainable bioreactor processes^10,18,31^.

**Scheme 1:**
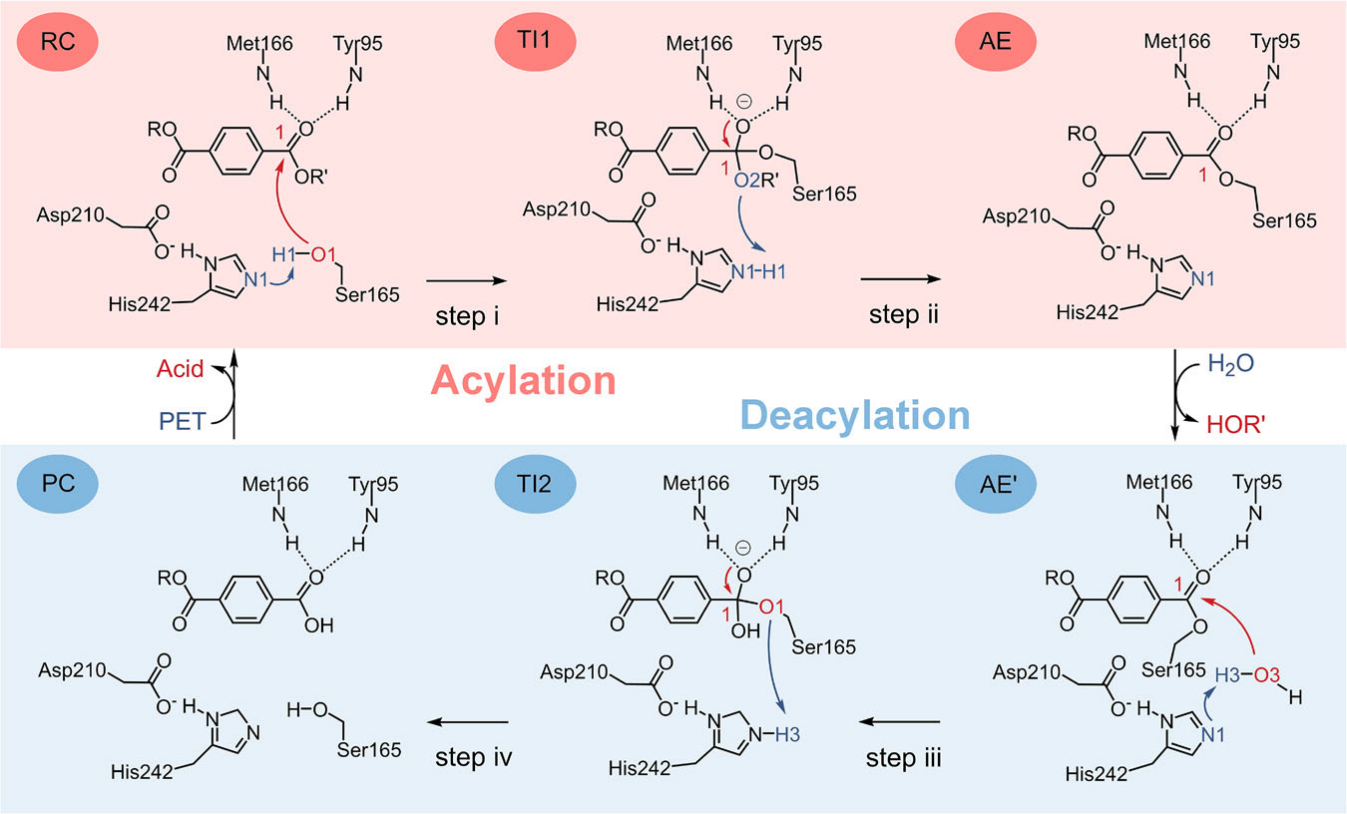
Proposed catalytic mechanism of LCC^ICCG^ toward PET. The red and blue shadings represent the acylation and deacylation stages, respectively.

It is widely accepted that fully deciphering the biocatalytic mechanism of PET is vital for the rational design of superior enzymes^32,33^. The acylation-deacylation mechanism of PET hydrolases has been well established, wherein acylation and deacylation refer to the acylation and deacylation of the catalytic serine^31,34–40^. It has been proposed that LCC^ICCG^ also followed this classical acylation-deacylation mechanism (Scheme 1)^41–43^. The acylation stage is initiated by the nucleophilic attack of Ser165 to the carboxyl carbon of the PET substrate and leads to the formation of an acyl-enzyme intermediate. Subsequently, an active-site water attacks the acyl-enzyme intermediate and the enzyme is deacylated to its initial state during the deacylation stage. Additionally, it has been proposed that PET hydrolases bind nonspecifically to the substrate, forming either a productive complex that enables PET hydrolysis or an unproductive complex incapable of catalyzing this reaction^44,45^. However, little is known about the product release process.

Enzyme built-in internal electric fields (IEFs) generated by protein scaffolds have been proposed as a vital driving force in enzyme catalysis^46–49^. This concept is theoretically supported by findings that IEFs preferentially stabilize the transition state over the reactant state^50–53^. Experimental Stark spectroscopy verified the existence of enzyme IEFs and confirmed their contribution to reaction rates^54–56^. Recently, many computational studies focus on quantifying the preorganized IEFs of enzymes (e.g. E:S complex), leaving the dynamic behavior of IEFs along the catalytic process largely unexplored. Considering that an energy barrier involves both the transition state and reactant/intermediate, examining the dynamic fluctuations between different states can be crucial.

To sum up, the PET hydrolysis mechanism remains unclear in several aspects: (i) It is controversial whether the acylation or deacylation stage follows a one- or two-step mechanism. (ii) It remains unclear which process is rate-determining when substrate binding and product release are considered. (iii) The main driving forces and the contribution of built-in IEFs behind enzymatic PET depolymerization are largely unexplored. To shed light on these knowledge gap, we leveraged extensive quantum mechanics/molecular mechanics molecular dynamics (QM/MM MD) simulations to study the hydrolysis of PET by LCC^ICCG^, with PET hexamer as a showcase. The whole hydrolysis process, including PET hexamer binding, acylation, (mono(2-hydroxyethyl) terephthalate)_2_ (MHET_2_) release, deacylation, and final product release, was systematically studied. We also explored the IEF fluctuations during the catalytic process and identified its role in enzyme catalysis. Machine learning methods like random forest, multilayer perceptron, and extreme gradient boosting were used to analyze the relationships of the IEF along different orientations.

## Results and discussion

### PET hexamer binding into the active site occurs far below the diffusion limit

**Fig. 1:**
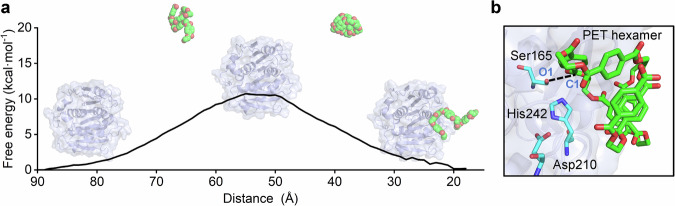
Binding of the PET hexamer into the active site of the LCCICCG variant. **a** Free energy profile of the PET hexamer binding to the active site. LCC^ICCG^ variant and PET chain are shown in cartoon and spheres, respectively. The free energy profile was obtained from umbrella sampling simulations performed at the MM level. The X-axis is labeled as “Distance”, which represents the center-of-mass distance between the LCC^ICCG^ and the PET hexamer and serves as the reaction coordinate for the binding process. **b** Close-up view of the LCC^ICCG^ active site bound to the PET hexamer substrate. The catalytic triad and PET hexamer are shown in blue and green sticks, respectively.

Like many other reported PET hydrolases^12,14–17^, LCC^ICCG^ features an active site on its surface. This architecture is conducive to efficient PET access and subsequent rapid depolymerization. It has been proposed that interfacial catalysis plays a key role during PET depolymerization while there are plenty of enzyme engineering strategies root on altering enzyme’s surface charges^9,57–59^. However, the free energy profile of PET chain binding into the enzyme’s active site remains largely unclear. Here, with extensive umbrella sampling (>700 ns), we determined the free energy profile of PET hexamer binding to its active site. We highlight here that rather than the frequently simulated PET dimer, we used a PET hexamer, which better represents realistic flexible PET plastic segments, for the subsequent simulations. The calculated energy barrier for PET hexamer binding into the active site of LCC^ICCG^ is 10.7 kcal mol^−1^ (Fig. 1a and S1). This energy barrier may arise from conformational changes in the PET hexamer required for fitting into the active site, as well as its interactions with amino acid residues surrounding the binding cleft (Figs. S2–S4).

According to the Eyring equation, the energy barrier corresponds to a rate constant of ~10^5 ^s^−1^ at 343 K, which is far below the diffusion limit of small chemicals (~10^9 ^M^−1^ s^−1^) or large proteins (~10^7–8^ M^−1^ s^−1^) in water. We show that despite the active site of LCC^ICCG^ being located on its surface, PET hexamer binding is not diffusion controlled. Meanwhile, the calculated rate constant is higher than the experimentally determined rates of ester hydrolysis in PET plastic (about 0.1–10 s^−1^)^60–62^, which indicates that enzyme-catalyzed chemical processes or product release might be the rate-determining step. To explore the binding orientations of the PET hexamer at the active site, we performed extensive MD simulations at 343 K. Only one of the six PET units interacts tightly with LCC^ICCG^ while the remaining five units interact rather loosely with the active site (Fig. 1b and S5), which is consistent with previous studies on PET substrate binding^63^. The productive binding state is mainly characterized by two criteria: (i) PET binds in the *Si*-face mode (Fig. S6), and (ii) the C1–O1 distance is ≤4 Å. Our previous studies demonstrated that *Si*-face binding generally leads to higher catalytic activity^53,64^.

### Stepwise enzymatic hydrolysis mechanism

**Fig. 2:**
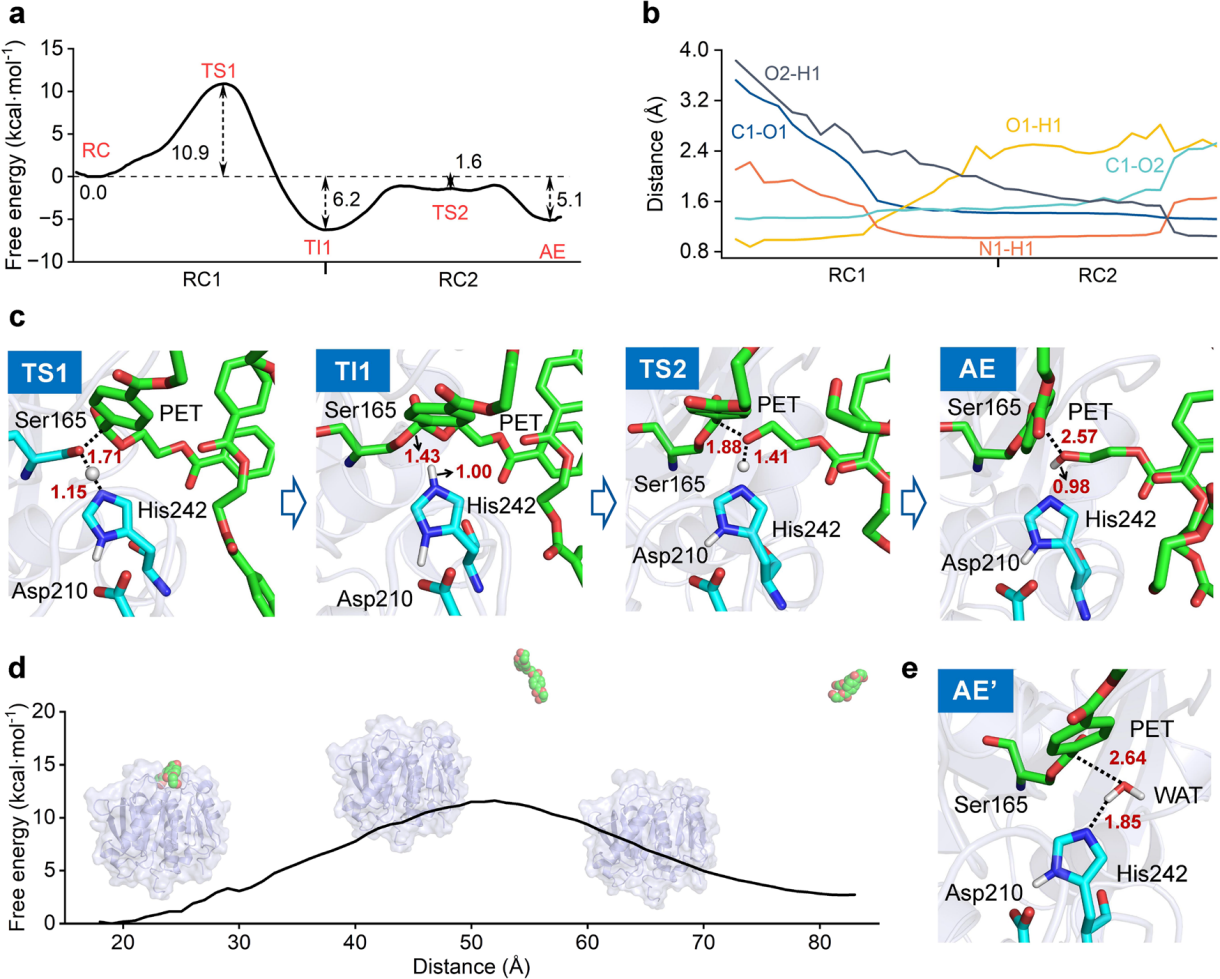
Acylation mechanism of PET hydrolysis by the LCCICCG variant. **a** Free energy profile for the acylation stage. The values of the free energy are given. RC1 and RC2 represent the reaction coordinates defined by CV_1_ and CV_2_, respectively, and the unit is Å. **b** Evolution of the key active site distances along the reaction coordinates. Average values calculated based on the last 15 ps simulation data. **c** Representative structures of the key states along the reaction coordinates for the acylation stage. The catalytic triad and PET hexamer are shown in cyan and green sticks, respectively. **d** Free energy profile of the departure of MHET_2_ from the active site. LCC^ICCG^ variant and MHET_2_ are shown in cartoon and spheres, respectively. The free energy profile was obtained from umbrella sampling simulations performed at the MM level. The *X*-axis is labeled as “Distance”, which represents the center-of-mass distance between LCC^ICCG^ and the MHET_2_ and serves as the reaction coordinate for the departure of the MHET_2_ from the active site. **e** Representative structures of AE’.

After formation of the Michaelis complex (E + S → E:S), the enzyme is ready to convert substrate to product through chemical processes. As for LCC^ICCG^ and other hydrolases, a cascade of acylation and deacylation stages has been well-established^32,65^. However, it is still controversial whether the acylation or deacylation stage follows the one- or two-step mechanism. This is partly because the one-step mechanism was determined directed via semi-empirical QM/MM simulations while two-step mechanism was established with multiple potential energy surface (multi-PES) DFT/MM calculations^43,66,67^ or DFT corrected semi-empirical QM/MM simulations^41,42^. Here, we leverage QM/MM MD simulations at M06-2X/6-31 G(d)//MM level to investigate the controversial hydrolysis mechanism (Scheme 1). As shown in Fig. 2a, the calculated free energy profile clearly demonstrates that the acylation stage follows a two-step mechanism. The transition states of each elementary step were validated using committor analysis (Fig. S7). The free energy barrier of the first step (step i) is 10.9 kcal mol^−1^, while that of the second step (step ii) is 4.6 kcal mol^−1^, which is lower than those obtained from other theoretical studies^41,42^. This difference may originate from differences in QM calculation methods and model substrate size^68,69^. It is interesting to show that step ii exhibits a relatively flat plateau transition state. After formation of acyl-enzyme intermediate (AE), the leaving group MHET_2_ gradually departs from the active site to the bulk solvent with an energy barrier of 11.6 kcal mol^−1^ (Fig. 2d and S8). Besides, the final state is 2.7 kcal mol^−1^ higher in energy than initial state, indicating that the departure of MHET_2_ is not a spontaneous process. However, the entire acylation process exhibits an overall energy barrier of 10.9 kcal mol^−1^ and a reaction energy of −5.1 kcal mol^−1^.

The two-step character of the acylation stage is also supported by key variations in parameters along the reaction coordinate (Fig. 2b, c). In the reactant complex (RC), the nucleophilic Ser165 is well oriented for nucleophilic attack on the carboxyl carbon (O1–C1: 3.5 ± 0.2 Å), and hydrogen bonds form between the carboxyl oxygen and the backbone amines of Tyr95 and Met161 in the oxyanion hole. The step i corresponds to the proton transfer from Ser165 to His242, with the N1–H1 distance decreasing from 1.9 ± 0.2 to 1.0 ± 0.0 Å (Figs. S9–S11). This is followed by nucleophilic attack by O1 on the carboxyl carbon (C1) of the PET hexamer, which leads to the formation of a tetrahedral intermediate (TI1). During step i, transition state 1 (TS1) and TI1 seem to be stabilized by the oxyanion hole. The distances for O4-H6 and O4-H7 shorten from 1.9 ± 0.2 to 1.6 ± 0.1 Å and 3.3 ± 0.5 to 1.9 ± 0.1 Å, respectively. In step ii, the cleavage of the C1–O2 bond (from 1.5 ± 0.1 to 2.6 ± 0.1 Å) is accompanied by a processive shortening of the distance between the proton H1 and O2 (from 2.0 ± 0.1 to 1.0 ± 0.0 Å), resulting in the formation of the acyl-enzyme intermediate (Figs. S12–S14). With the leaving group diffusing into the solvent, its position is occupied by a water molecule from the bulk solvent (AE’) (Fig. 2e). These key structural variations are similar to those described in the previous study^41,42^.

**Fig. 3:**
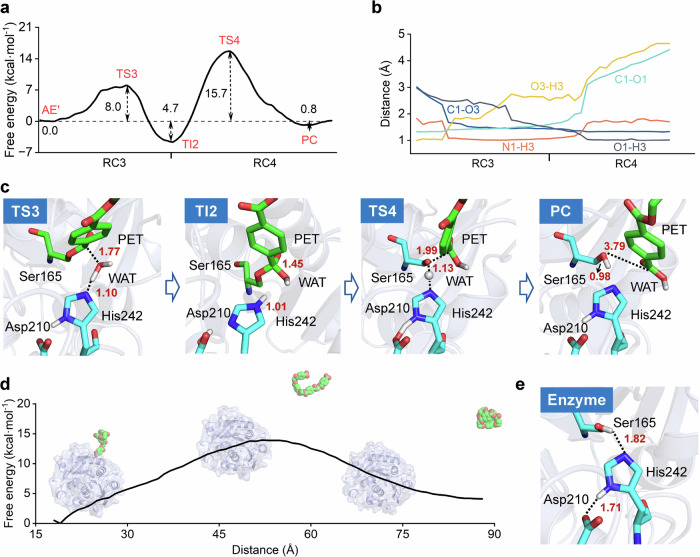
Deacylation mechanism of PET hydrolysis by the LCCICCG variant. **a** Free energy profile for the deacylation stage. The values of the free energy are given. The RC3 and RC4 represent the defined reaction coordinates by CV_3_ and CV_4_, respectively, and the unit is Å. **b** Evolution of the key active site distances along the reaction coordinates. Average values calculated based on the last 15 ps simulation data. **c** Representative structures of the key states along the reaction coordinates for the deacylation stage. The catalytic triad and PET chains are shown in cyan and green sticks, respectively. **d** Free energy profile of the departure of MHET_4_ from the active site. The enzyme and MHET_4_ are shown in cartoon and spheres, respectively. The free energy profile was obtained from umbrella sampling simulations performed at the MM level. The *X*-axis is labeled as “Distance”, which represents the center-of-mass distance between the LCC^ICCG^ and MHET_4_ and serves as the reaction coordinate for the departure of the product MHET_4_ from the active site. **e** Active site of the apo enzyme.

Similarly, the deacylation process also follows a two-step mechanism (Fig. 3). The free energy barriers for step iii and step iv are 8.0 and 20.4 kcal mol^−1^, while the free energy barrier for the product release is 13.9 kcal mol^−1^ (Figs. 3a, d and S15). Clearly, step iv has the highest energy barrier and is identified as the rate-determining step for deacylation. Structurally, an active-site water molecule is positioned close enough to the acyl-enzyme intermediate in AE’ (C1–O3: 3.1 ± 0.2 Å and N1–H3: 1.5 ± 0.0 Å) to trigger the nucleophilic attack and proton transfer reactions (step iii), generating a tetrahedral intermediate (TI2) (Figs. 3b, c, and S16–S17). The oxyanion hole hydrogen bond variation was similar to the acylation stage, which shortens from AE’ to TI2 (O4-H6 from 1.9 ± 0.2 to 1.6 ± 0.1 Å and O4-H7 from 2.1 ± 0.2 to 1.7 ± 0.1 Å), and increases from TI2 to product complex (PC) (O4-H6 from 1.6 ± 0.1 to 2.2 ± 0.2 Å and O4-H7 from 1.7 ± 0.1 to 4.2 ± 0.2 Å). This further confirms the stability role of the oxyanion hole in the transition state and intermediate. In step iv, the final PC is generated through cleavage of the ester bond (C1–O1: from 1.5 ± 0.1 to 4.4 ± 0.1 Å), accompanied by proton transfer from His242 to Ser165 (O1–H3: from 1.8 ± 0.1 to 1.0 ± 0.1 Å) (Fig. 3e and S18-S19).

**Fig. 4:**
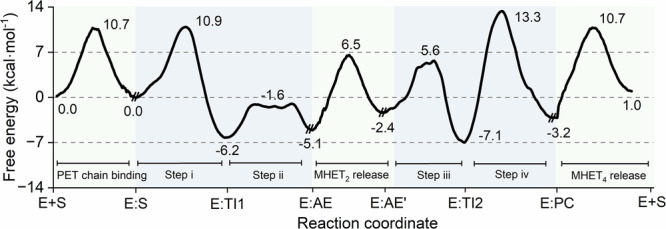
The global free energy profile for the hydrolysis of PET by the LCCICCG variant. The PET chain binding, MHET_2_ release, and MHET_4_ release were calculated at the MM level, while steps i– iv were calculated at the QM/MM level.

As a result, the global free energy profile of the LCC^ICCG^-catalyzed cycle for the hydrolysis of an ester bond in the PET hexamer is established (Fig. 4). The full cycle involves PET chain binding (E + S → E:S), acylation (E:S → E:TI1 → E:AE), MHET_2_ release (E:AE → E:AE’), deacylation (E:AE’ → E:TI2 → E:PC), and MHET_4_ release (E:PC → E + S). The energy barriers for substrate entrance (10.7 kcal mol^−1^) and product release (13.9 kcal mol^−1^) are significantly lower than the rate-determining step during the deacylation process (E:TI2 → E:PC), which requires a free energy barrier of 20.4 kcal mol^−1^. The result is in good accordance with the experimentally determined rate constant (0.3–1.6 s^−1^) of LCC^ICCG^-catalyzed ester bond hydrolysis in PET^61,62^, which corresponds to an energy barrier of 17.1–18.1 kcal mol^−1^. Similar kinetics for *Is*PETase-catalyzed PET hydrolysis using a bulky assay (~0.8 s^−1^) and single-molecule microscopy (~0.37 s^−1^) were found^70,71^.

### Dynamic electric field shifts catalyze PET hydrolysis

**Fig. 5:**
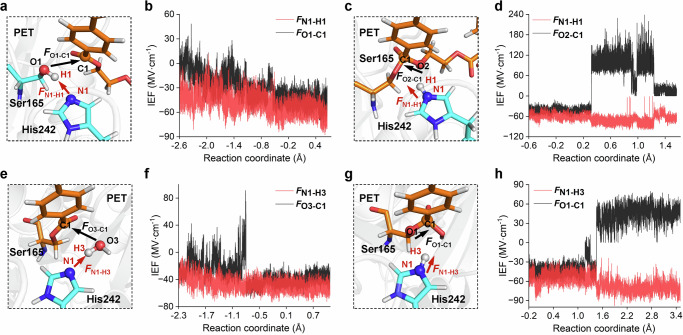
Internal electric fields generated by the enzyme environment. **a** Definition of the positive direction along the O1–C1 and N1–H1 axes. **b** The calculated electric fields along the O1–C1 and N1–H1 axes along the reaction coordinate in step i. **c** Definition of the positive direction along the O2–C1 and N1–H1 axes. **d** The calculated electric fields along the O2–C1 and N1–H1 axes along the reaction coordinate in step ii. **e** Definition of the positive direction along the O3–C1 and N1–H3 axes. **f** The calculated electric fields along the O3-C1 and N1–H3 axes along the reaction coordinate in step iii. **g** Definition of the positive direction along the O1–C1 and N1–H3 axes. **h** The calculated electric fields along the O1–C1 and N1–H3 axes along the reaction coordinate in step iv.

**Fig. 6:**
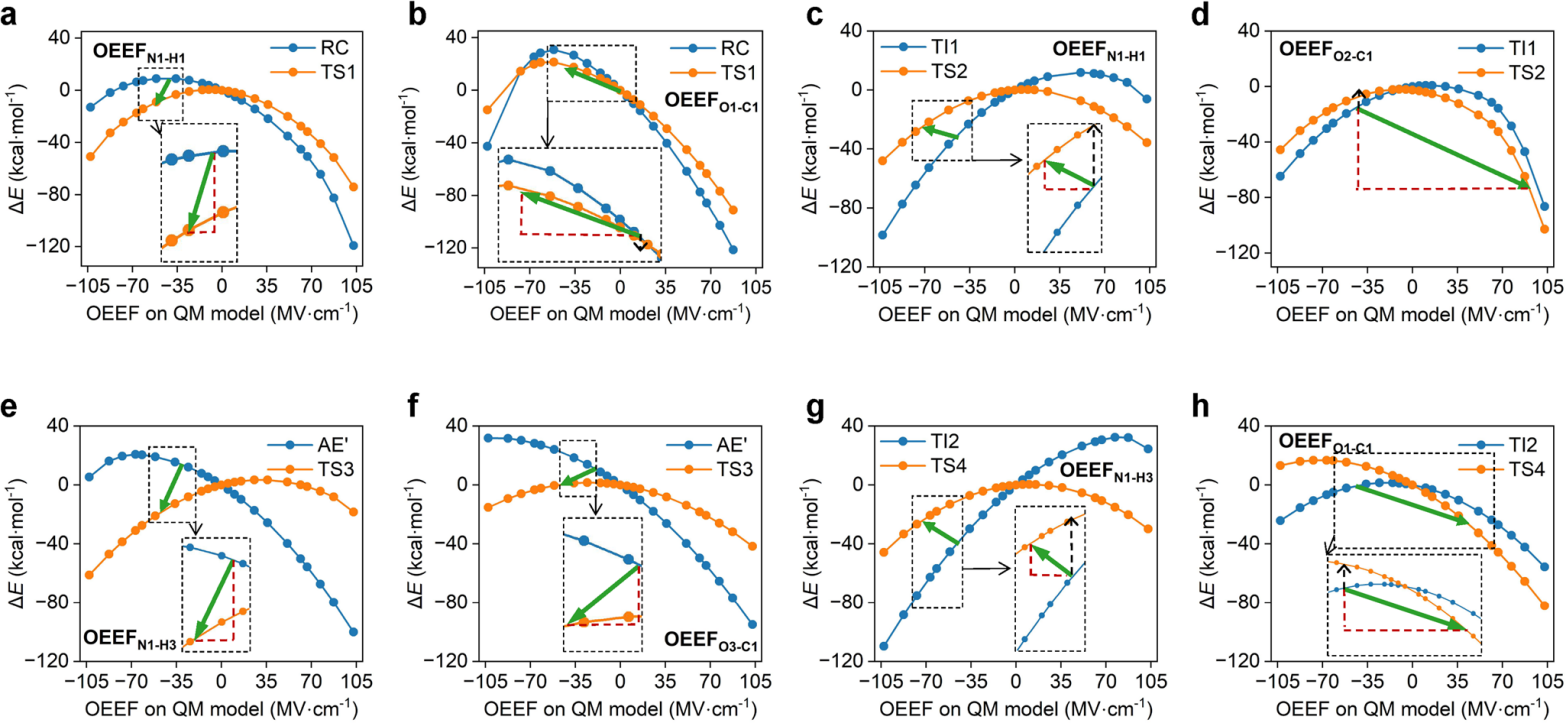
Effects of OEEFs along the reaction axes. **a**, **b** The effects of OEEFs along the N1–H1 (**a**) and O1–C1 (**b**) axes on the energies of the reactant (RC) and transition state (TS1) in step i. **c**, **d** The effects of OEEFs along the N1–H1 (**c**) and O2–C1 (**d**) axes on the energies of the reactant (TI1) and transition state (TS2) in step ii. **e**, **f** The effects of OEEFs along the N1–H3 (**e**) and O3-C1 (**f**) axes on the energies of the reactant (AE’) and transition state (TS3) in step iii. **g**, **h** The effects of OEEFs along the N1–H3 (**g**) and O1–C1(**h**) axes on the energies of the reactant (TI2) and transition state (TS4) in step iv.

To estimate the dynamic fluctuations between different reaction states (reactant and transition state), enzyme built-in internal electric fields (IEFs) along the LCC^ICCG^-catalyzed hydrolysis process were analyzed, and its impact on the free energy profile was evaluated. For step i (RC → TI1), the N1–H1 and O1–C1 distances are essential for characterizing the reaction process. (Fig. 5a). We observed that the IEFs along the N1–H1 direction (denoted as *F*_N1–H1_) fluctuates from −37.1 ± 11.1 to −52.5 ± 10.2 MV cm^−1^ (RC → TS1), while that along O1–C1 (*F*_O1–C1_) fluctuates from −3.7 ± 10.7 to −41.7 ± 6.6 MV cm^−1^ (Fig. 5b). The observed significant fluctuations provide evidence that the average IEFs along the reaction pathway are not constant. Similar results have also been reported in another enzymatic catalytic system^72^. To evaluate the effect of dynamic shift in IEFs on the reaction energy barrier, OEEFs analysis along the O1–C1 (OEEF_O1–C1_) and N1–H1 (OEEF_N1–H1_) axes were performed. For instance, for a given negative *F*_N1–H1_ value, it stabilizes the transition state (TS1) more than the reactant state (RC) (Fig. 6a). For the enzyme built-in IEF (e.g. −37.1 ± 11.1 MV cm^−1^), *F*_N1–H1_ already promotes catalysis by lowering the energy barrier by 9.4 kcal mol^−1^ if RC and TS1 shares the same IEFs. However, since average *F*_N1–H1_ dynamically shifts from −37.1 ± 11.1 (RC) to −52.5 ± 10.2 MV cm^−1^ (TS1), *F*_N1–H1_ further stabilizes TS1 than RC and thus accelerates catalysis. It reduces the energy barrier by an additional value of 5.7 kcal mol^−1^. In contrast, fluctuations in *F*_O1–C1_ destabilize TS1 and do not facilitate the nucleophilic attack.

In step ii (TI1 → TS2), *F*_N1–H1_ shifts from −53.8 ± 6.5 to −73.3 ± 7.7 MV cm^−1^, while *F*_O2–C1_ changes from −42.4 ± 6.5 to 93.8 ± 12.8 MV cm^−1^ (Fig. 5c, d). Step ii involves the gradual cleavage of the C1–O2 bonds, which induces changes in the active-site structure and subsequently affects the whole protein scaffold. The significant fluctuations in *F*_O2–C1_ may originate from changes in the relative positioning between the enzyme and the substrate (Fig. S20). Combined with the OEEFs results (Fig. 6c, d), we conclude that *F*_N1–H1_ slightly hinders N1–H1 cleavage and increases the energy barrier. This aligns with the “beneficial” role of *F*_N1–H1_ in step i, as in step ii, histidine donates a proton rather than abstracting one. However, we argue that the enzyme has already attempted to facilitate N1–H1 cleavage through a dynamic electric field shift: if there were no dynamic shift, the energy barrier would increase by ~13.0 kcal mol^−1^ rather than ~2.0 kcal mol^−1^. More interestingly, a significant dynamic electric field shift in *F*_O2–C1_ has completely reversed the IEFs while ultimately enhancing the O2–C1 cleavage process.

The IEFs (*F*_N1–H1_ and *F*_O3–C1_) in step iii are similar to those in step i, as indicated in Fig. 5e, f. The calculated OEEF results are also comparable (Fig. 6e, f). One key difference is that both *F*_N1–H1_ and *F*_O3–C1_ facilitate the nucleophilic attack of the water molecule as well as the proton transfer. Meanwhile, the IEFs (*F*_N1–H1_ and *F*_O1–C1_) in step iv resemble those in step ii, where a significant *F*_O1–C1_ fluctuation was also observed (Fig. 5g, h, and S20). Results from OEEFs calculations (Fig. 6e, f) once again confirm the beneficial role of a dynamic electric field shift. Besides, robust random forest, multilayer perceptron, and extreme gradient boosting models were established to analyze the relationships of the IEFs along different reaction axes (Figs. S21–S29). *F*_N1–H1_ and *F*_O1–C1_ exhibit a high correlation during the acylation stage, while *F*_O1–C1_ and *F*_C1–O4_ show a strong correlation during the deacylation stage. We propose that LCC^ICCG^ may play a role in modulating the IEFs along different reaction axes during the catalytic process. In summary, the above results clearly show that a dynamic electric field shift is a common feature in enzymatic PET hydrolysis and may also occur in other enzymatic processes.

## Conclusions

Here, we leverage extensive QM/MM MD simulations to investigate the controversial PET hydrolysis mechanism. The global free energy profile of the LCC^ICCG^-catalyzed hydrolysis cycle toward the PET hexamer is established, and the results support a two-step mechanism. Both PET hexamer binding into the active site and product release exhibit unexpectedly high free energy barrier (10.7–13.9 kcal mol^−1^), with corresponding rates significantly below the diffusion limit. The rate-determining step of PET hydrolysis is the fourth step (step iv), with an estimated free energy barrier of 20.4 kcal mol^−1^, consistent with the experimentally determined rate constant (0.3–1.6 s^−1^). We observed that the enzyme built-in IEFs shift dynamically during the catalysis process, with the transition state and reactant/intermediate exhibiting different average field strengths. The effect of this “dynamic shift” in the IEFs was clarified through the application of OEEFs in a QM cluster model, which demonstrated that this shift actively facilitates PET hydrolysis by reducing the energy barrier. These new insights underscore the role of IEF dynamic shift and may contribute to the global optimization of enzyme performance.

## Methods

### System preparation

The high-resolution X-ray crystal structure of the LCC^ICCG^ variant (F243I/D238C/S283C/Y127G) was obtained from the Protein Data Bank (PDB ID: 6THT, resolution: 1.14 Å)^27^. The mutated catalytic residue Ser160Ala was reverted to serine using Chimera software^73^. The protonation states of titratable residues at physiological pH (pH = 7.0) were determined based on the calculated p*K*_a_ values by PROPKA3.0 software^74^ (Table S1) and by careful visual inspection of the local hydrogen bond networks. All lysine and arginine residues were protonated, while all glutamic acid and aspartic acid residues were deprotonated. His207 was protonated at the δ position, while His77, His129, His156, His183, and His256 were protonated at the ε position. A PET hexamer (six repeat units, charge −1) was used as the model substrate. It is sufficiently large to capture key interactions within the enzyme active site and to investigate the catalytic reaction mechanism^63^. The binding positions of the model substrate within the active site of the LCC^ICCG^ variant were obtained using Autodock Vina software^75^. Charges for both the protein and the substrate were assigned using the Gasteiger method^75^. The oxygen atom (O1) of the catalytic residue Ser165 was selected as the center of the grid box, and the grid dimensions along the *X*-, *Y*-, and *Z*-axes were set to 30, 30, and 30, respectively. The conformation with the shortest C1–O1 distance was selected as the optimal docking pose for subsequent classical MD simulations. Although one binding pose was selected as the starting structure, the PET substrate was allowed to adjust its pose freely during the MD simulation. The substrate PET hexamer was parametrized according to the General AMBER Force Field (GAFF) using AM1-BCC charges^76,77^. The system was prepared using the tleap module of AMBER24 software^78^, and the enzyme was described with the AMBER ff14SB force field^79^. The system was solvated in a rectangular TIP3P water box with a buffer region of 12 Å from the enzyme:substrate complex^80^. Five chlorine ions were added to neutralize the total charge of the system. The resulting system comprised 36399 total atoms, including 3848 protein atoms, 134 PET atoms, 10804 water molecules, and 5 counterions (Fig. S30).

### Classical MD simulations

All MD simulations were performed using the AMBER24 software package. The entire system was minimized using 2000 steps of the steepest descent algorithm followed by the conjugate gradient method to avoid clashes. Initially, the enzyme-substrate complex was held with a positional restraint force constant of 50 kcal mol^−1^ Å^-2^, while the water molecules and counterions were minimized. Then, the entire system, expect for the active site residues, was minimized. Finally, the entire system was fully minimized without any restraints. The LCC^ICCG^ exhibits high activity at ~70 °C^27^. The temperature and pressure of the system were set to 343.15 K and 1 bar using the Langevin thermostat^81^ and the Berendsen barostat^82^, respectively. Afterward, 10 ns of equilibration were performed with the NPT ensemble. Periodic boundary conditions were applied. The SHAKE algorithm was used to constrain all bond lengths involving hydrogen atoms. The particle-mesh Ewald (PME) method was employed to calculate long-range electrostatic interactions with a cutoff distance of 12 Å^83^. Three independent 500 ns MD simulations were performed with a 2 fs timestep (Figs. S31,S32).

### QM/MM calculations

A representative conformation was extracted from the classical MD trajectory as the starting point for QM/MM simulations, which represents the dominant conformational population observed in the MD trajectory (Figs. S33, S34). All QM/MM calculations were performed using the AMBER12 program interfaced with Q-Chem^84,85^. For the acylation stage, the QM region contained the side chains of the Ser165, His242, and Asp210 (catalytic triad), the side chain of Tyr95 and Met161 (oxyanion hole), and a portion of the PET substrate (Fig. S35). The entire QM region for the acylation step consisted of 90 atoms. The charge of the QM region was set to −1. For the deacylation stage, an acyl-enzyme configuration was taken from the acylation reaction trajectory. The MHET_2_ product was removed, and the enzyme-ligand complex was re-solvated and neutralized. The minimization and equilibration procedures were the same as in the acylation stage. The QM region for the deacylation step consisted of 69 atoms (Fig. S35). The charge of the QM region was set to −1. The M06-2X functional^86^ with the 6-31 G(d) basis set was applied to the QM region, while the AMBER ff14SB force field was used for the MM region. Previous studies have demonstrated that the M06-2X functional is suitable for investigating the enzyme-catalyzed PET reaction mechanism^41,42,67,87^. The QM/MM boundary was treated with the improved pseudo-bond approach^88,89^. The time step was set to 1 fs. All other parameters remain as described for the classical MD simulations. The system was first subjected to QM/MM energy minimization, followed by 20 ps QM/MM MD equilibration. The minimum energy path was determined using the reaction coordinate driving method, with at least three forward and backward reaction path scans to obtain smooth energy profiles^90–93^. The MM region was further equilibrated by 500 ps simulations, while the QM region was kept fixed. The resulting conformations served as starting structures for subsequent QM/MM umbrella sampling (US) simulations. The collective variables (CVs), CV_1_ = (d_1_-d_2_), CV_2_ = (d_3_-d_4_), CV_3_ = (d_5_-d_6_), and CV_4_ = (d_7_-d_8_), were used to define the reaction coordinates describing the chemical mechanism of LCC^ICCG^ (Figs. S36–S39). Shortening of d_2_(C1–O1) and d_6_(C1–O3) implies the formation of C–O bonds, while elongation of d_3_(C1–O2) and d_7_(C1–O1) shows the cleavage of C–O bonds. Similarly, the elongation of d_1_(O1–H1) and d_5_(O3–H3) indicates the proton transfer toward the catalytic residue His242 while shortening of d_4_(O2–H1) and d_8_(O1–H3) implies the proton transfer from the catalytic residue His242.

### Umbrella sampling simulations

QM/MM MD umbrella sampling simulations have been successfully applied in studying enzymatic processes, such as thymidine kinase, Creatininase, and Chitinase^94–101^. Here, the free energy profile along the reaction coordinate for each chemical step was explored with umbrella sampling at the QM/MM level stated above^102^. For sampling along the reaction coordinates, the reaction coordinates were divided into space windows of different widths (Tables S2–S5). We should note that different umbrella sampling window widths were chosen to enhance overlap between neighboring windows and to improve sampling efficiency. The harmonic potentials applied to each window to ensure proper overlap of the umbrella sampling windows (Figs. S40–S43), and the values of the force constant were provided in Tables S2–S5. All umbrella sampling simulations were performed for 30 ps in each window: 36 windows for the catalytic triad-initiated nucleophilic attack, 34 windows for the C–O bond cleavage of substrate, 28 windows for the nucleophilic attack by the water molecule, and 38 windows for the C–O bond cleavage of acyl-enzyme intermediate. The free energy profiles were calculated based on the last 15 ps of simulation data employing the weighed histogram analysis method (WHAM)^103^, with 100 bootstrap data sets and a tolerance threshold of 0.00001 kcal mol^−1^. The estimated statistical uncertainty of the free energy is presented in Table S6. The convergence of the free energy profiles has been tested (Figs. S44–S47). The transition states of each elementary step were validated by committor analysis, and the method details are provided in the Supporting Information. Other details for the US simulations of the substrate binding and the release of product are also provided in the Supporting Information.

### Substrate binding and product release

Umbrella sampling at the MM level was used to calculate the free energy profiles for substrate binding, and the release of products MHET_2_ and MHET_4_. A single harmonic restraint with a force constant of 2.5 kcal mol^−1^ Å^−2^ was applied to the distance between the center of mass of the protein and that of the PET hexamer, MHET_2_, or MHET_4_, along the *x*, *y*, and *z* axes, respectively. Preliminary tests indicated that a force constant of 2.5 kcal mol^−1^ Å^-2^ ensured sufficient overlap between neighboring sampling window in the present system. The free energy profile of substrate PET hexamer binding to the enzyme active site was obtained by simulating the process of pulling the substrate out of the active site. The initial structure (bound state) was defined as the optimal binding pose obtained from molecular docking, whereas the final structure (unbound state) corresponds to a local minimum on the free energy profile. The umbrella sampling simulations were performed at intervals of 1 Å along the respective axis. The windows numbers used for each simulation are provided in Table S7. Each umbrella sampling window was run for 10 ns, and the final 5 ns were used to calculate the free energy profiles. The WHAM was used to calculate the free energy profile.

### IEF calculations and their effects

The IEF is defined as the electric field exerted by the whole protein residues except for the catalytic residues Ser165, His242, and Asp210. The strength and direction of the IEFs along the reaction axes were calculated for the last 15 ps QM/MM MD umbrella sampling simulations using Coulomb’s law^52,104,105^:

1 $${\vec{F}}_{{{{\rm{IEF}}}}}=\frac{1}{4{{{\rm{\pi }}}}{{{{\rm{\varepsilon }}}}}_{0}}{\sum }_{i}\frac{{q}_{i}}{{\left|{\vec{r}}_{0}-{\vec{r}}_{i}\right|}^{2}}\cdot \frac{{\vec{r}}_{0}-{\vec{r}}_{i}}{\left|{\vec{r}}_{0}-{\vec{r}}_{i}\right|}$$

Here, ${q}_{i}$ and $\vec{r}$ present the charge and position of atom *i* in the protein residue. Charge parameters were obtained from the AMBER ff14SB force field.$\,{\vec{r}}_{0}$ is the midpoint of the reaction axes. The effects of IEF were evaluated using gas-phase QM calculations. The QM models were identical to the QM region in the QM/MM MD simulations. Oriented external electric fields (OEEFs) aligned with the reaction axes were added to the QM models for the reactant/intermediate and transition state geometries of each step. To maintain consistency with the QM/MM MD simulations, the energies were calculated at the M06-2X/6-31 G(d) level of theory. A larger basis set was also tested for energy calculations, and the results indicated that it did not affect the conclusions regarding the OEEF effects (Fig. S48).

### Deep learning models for internal electric field

Random Forest (RF), Multilayer Perceptron (MLP), and Extreme Gradient Boosting (XGBoost) were adopted to systematically compare the correlations and nonlinear fitting relationships of IEF governing enzymatic PET acylation and deacylation processes. The dataset comprised seven enzyme activity-related features (Table S8), standardized through z-score normalization and preprocessed using Shapley Additive Explanations (SHAP) to enhance interpretability. More details are provided in the Supporting information.

## Supplementary information

Supplemental Material

## Data Availability

All data supporting the paper are available within the article and Supplementary Information.
