# Supplementary material for: Dynamic shift of internal electric field accelerates enzymatic polyethylene terephthalate depolymerization
Source: Commun Chem. 2026 Jan 12;9:85. doi: 10.1038/s42004-026-01888-w (PMC12905295; doi:10.1038/s42004-026-01888-w)
Supplement: Supplementary file 1 — Supplemental Material [file 42004_2026_1888_MOESM1_ESM.pdf]

Supplementary Information

Dynamic Shift of Internal Electric Field Accelerates Enzymatic Polyethylene Terephthalate Depolymerization

Mingna Zheng[1], Jinfeng Chen[2], Weiliang Dong[3], Ren Wei [4], Jinyue Chen[1], Xiaowen, Tang[5]*, Qingzhu Zhang[1], Qiao Wang[1], Wenxing Wang[1], Guoqiang Wang[1]*, Yanwei Li[1]*

[1]Academician Workstation for Big Data in Ecology and Environment, Environment Research Institute, Shandong University, Qingdao, 266237, PR China

[2] Key Laboratory of Structural Biology of Zhejiang Province, School of Life Sciences, Westlake University, Zhejiang, 310024, PR China

[3]College of Biotechnology and Pharmaceutical Engineering, Nanjing Tech University, Nanjing, 211800, PR China

[4] Institute of Biochemistry, Department of Biotechnology & Enzyme Catalysis, University of Greifswald, D-17487, Greifswald, Germany

[5]Department of Medical Chemistry, School of Pharmacy, Qingdao University, Qingdao, 266071, PR China

*Corresponding authors.

Yanwei Li: E-mail: lyw@sdu.edu.cn

Guoqiang Wang: E-mail: 202188900012@sdu.edu.cn

Xiaowen Tang: E-mail: xwtang1219@qdu.edu.cn

Number of Page:66 (S1-S66)

Number of Figure:48 (Figure S1-S48)

Number of Table: 8 (Figure S1-S8)

Supplementary methods

Committor analysis for the validation of the transition state. We performed a committor analysis to validate the transition states of each elementary steps. Sixty representative structures with CV values corresponding to the maxima of the free energy profiles were selected, and these structures served as the starting point for the committor analysis. For each structure, we performed 100 fs unbiased MD simulation. A good TS configuration should exhibit approximately equal probabilities of evolving toward the reactants or products. Thus, the probability of the system evolving toward the reactants or products was calculated as the ratio of trajectories reaching each endpoint to the total number of simulations.

Model development and evaluation. Data preprocessing was carried out to ensure consistency and comparability across all features. Missing values were first addressed by removing outliers and imputing incomplete data points. Feature selection was then performed through correlation analysis and variance filtering to obtain the final seven-feature subset. Finally, z-score normalization was applied on a per-feature basis, where the mean of each feature was subtracted and the result divided by its standard deviation. This feature-wise normalization ensured that each enzyme-related metric contributed equally, regardless of its original units, and is the most appropriate method when features are measured on different scales. Random Forest (RF), Multilayer Perceptron (MLP), and Extreme Gradient Boosting (XGBoost) were adopted to systematically compare the correlations and nonlinear fitting relationships governing enzymatic PET acylation and deacylation. The dataset comprised seven enzyme activity-related features, standardized through z-score normalization and preprocessed using Shapley Additive Explanations (SHAP) to enhance interpretability. RF employed bootstrap aggregation with 100 decision trees (minimum leaf samples=1, Gini impurity splitting criterion) to mitigate multicollinearity, effectively capturing global interactions such as $F_{\mathrm{O1\text{-}C1}}/F_{\mathrm{N1\text{-}H1}}$ orbital synergies and the nonlinear influence of substrate amination on enzyme structural stability. MLP, structured as a fully

connected neural network with dual hidden layers (200-100 nodes, ReLU activation), modeled high-order kinetic nonlinearities (e.g., $F_{O3\text{-}C1}/F_{O3\text{-}H3}$ bond polarization coupling) using Adam optimization (initial learning rate=1×10^{-3}) to prevent overfitting. XGBoost, a gradient-boosted decision tree framework, integrated L1/L2 regularization and ensemble weak classifiers (CART trees, max depth=8, learning rate=0.05) to address high-dimensionality challenges in small-sample regimes, emphasizing critical factors like $F_{O1\text{-}H1}$-dependent enzyme conformational dynamics and substrate surface roughness thresholds. Model robustness was rigorously validated via five-fold cross-validation (mean squared error, MSE), while SHAP values systematically quantified feature importance across all methods, enabling mechanistic interpretation of enzyme-substrate binding energies, transition state energy barriers, and depolymerization product distributions. All computational workflows were implemented in Python 3.8 using Scikit-learn 1.0.2.

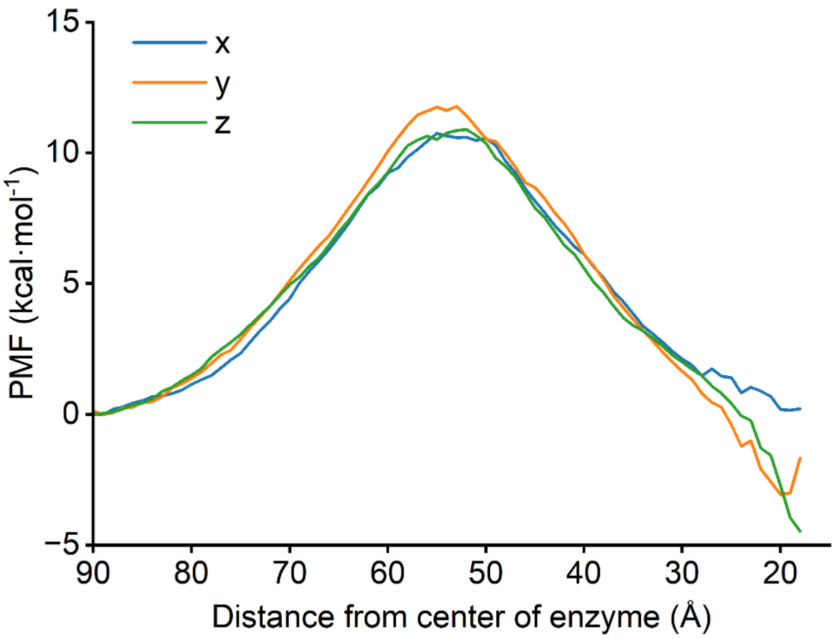

Figure S1. Free energy profiles of the PET hexamer binding the active site of the LCC^{ICCG} variant along the x, y, and z axes. Free energies were calculated at the MM level.

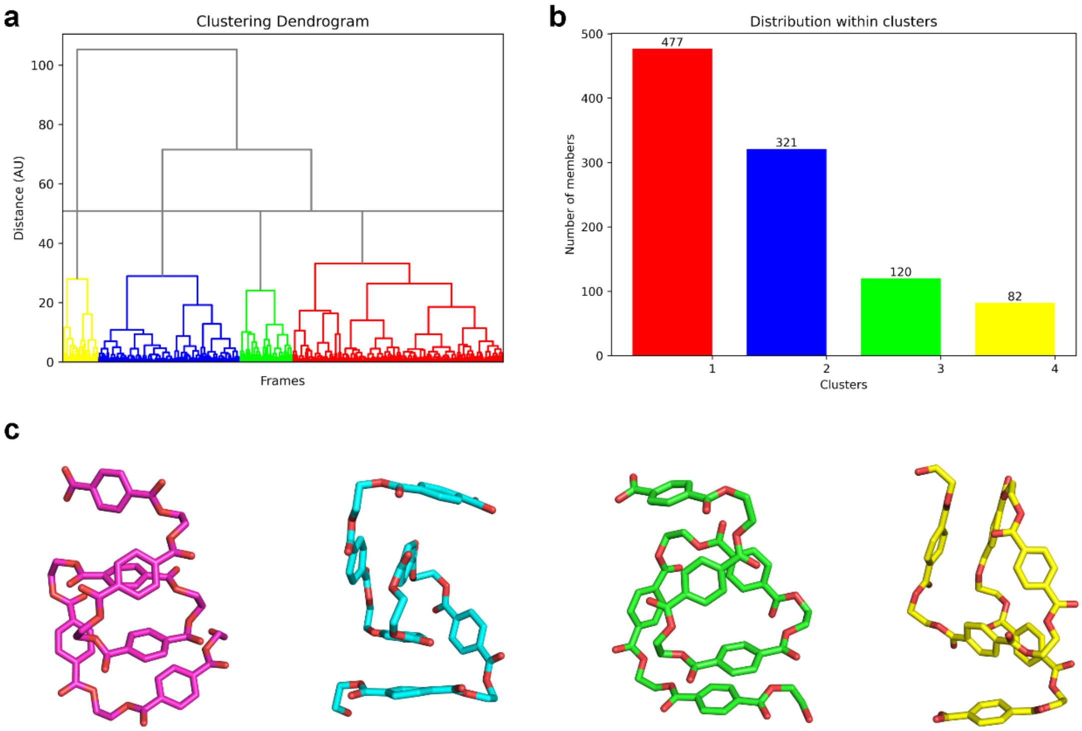

Figure S2. TTClust analysis of substrate conformations in the unbound state. **a.** Four clusters were identified in the unbound substrate state. **b.** Barplot numbers of frames within clusters. **c.** Representative substrate poses in the unbound state. The cluster color code is the same between the dendrogram, histogram and substrate poses. The heavy atoms of the PET hexamer were used for RMSD calculations.

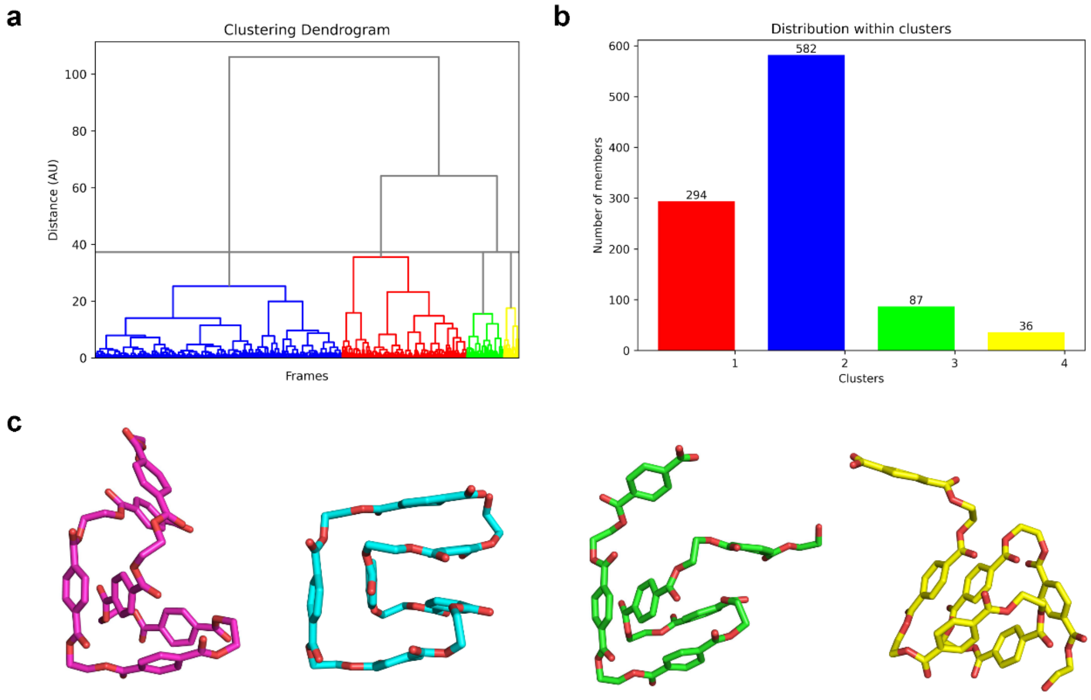

Figure S3. TTClust analysis of substrate conformations in the transition state. **a.** Four clusters were identified in the transition state. **b.** Barplot numbers of frames within clusters. **c.** Representative substrate poses in the transition state. The cluster color code is the same between the dendrogram, histogram and substrate poses. The heavy atoms of the PET hexamer were used for RMSD calculations.

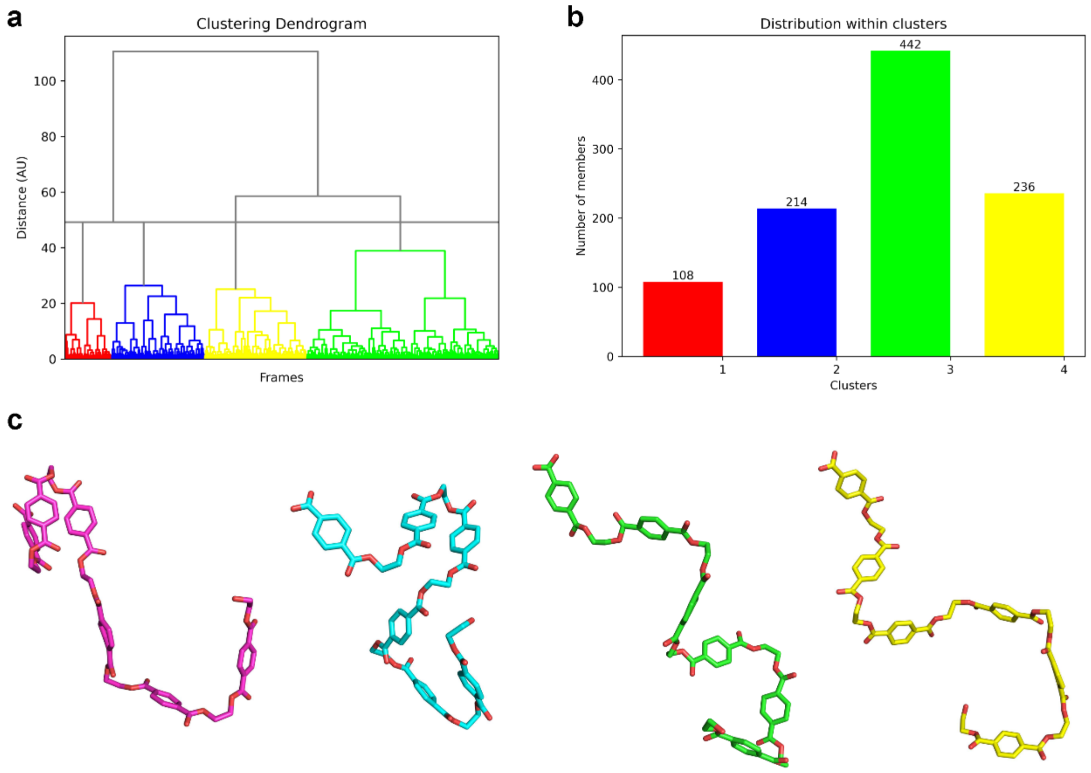

Figure S4. TTClust analysis of substrate conformations in the bound state. **a.** Four clusters were identified in the bound state. **b.** Barplot numbers of frames within clusters. **c.** Representative substrate poses in the bound state. The cluster color code is the same between the dendrogram, histogram and substrate poses. The heavy atoms of the PET hexamer were used for RMSD calculations.

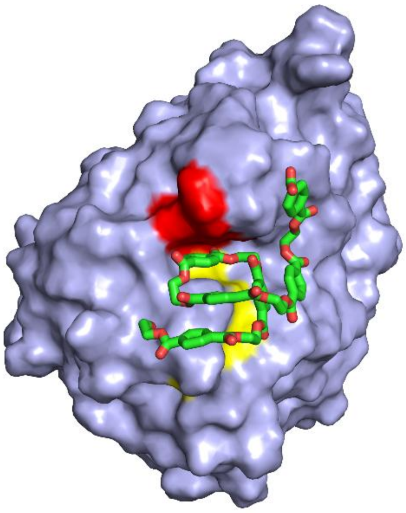

Figure S5. Michaelis complex of the LCC^{ICCG} variant. The enzyme is shown with a light blue surface, with the oxyanion hole and catalytic triad shown by yellow and red surface, respectively. The PET hexamer is shown with green sticks.

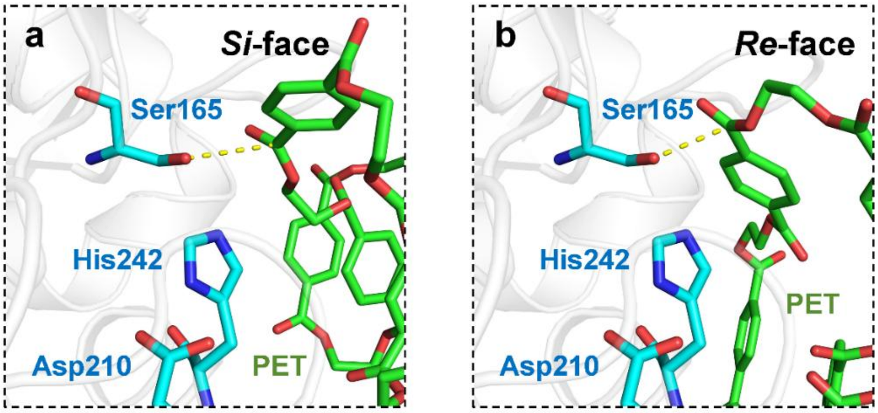

Figure S6. Different binding poses of PET substrate in the active site of the enzyme. **a.** *Si*-face binding. **b.** *Re*-face binding. Three substituents of the prochiral carbon of the PET substrate are ordered in a clockwise fashion, *Si*-face binding is defined as the serine nucleophilic attack on the prochiral carbon from the back face. The catalytic triad and the PET substrate are shown as blue and green sticks, respectively.

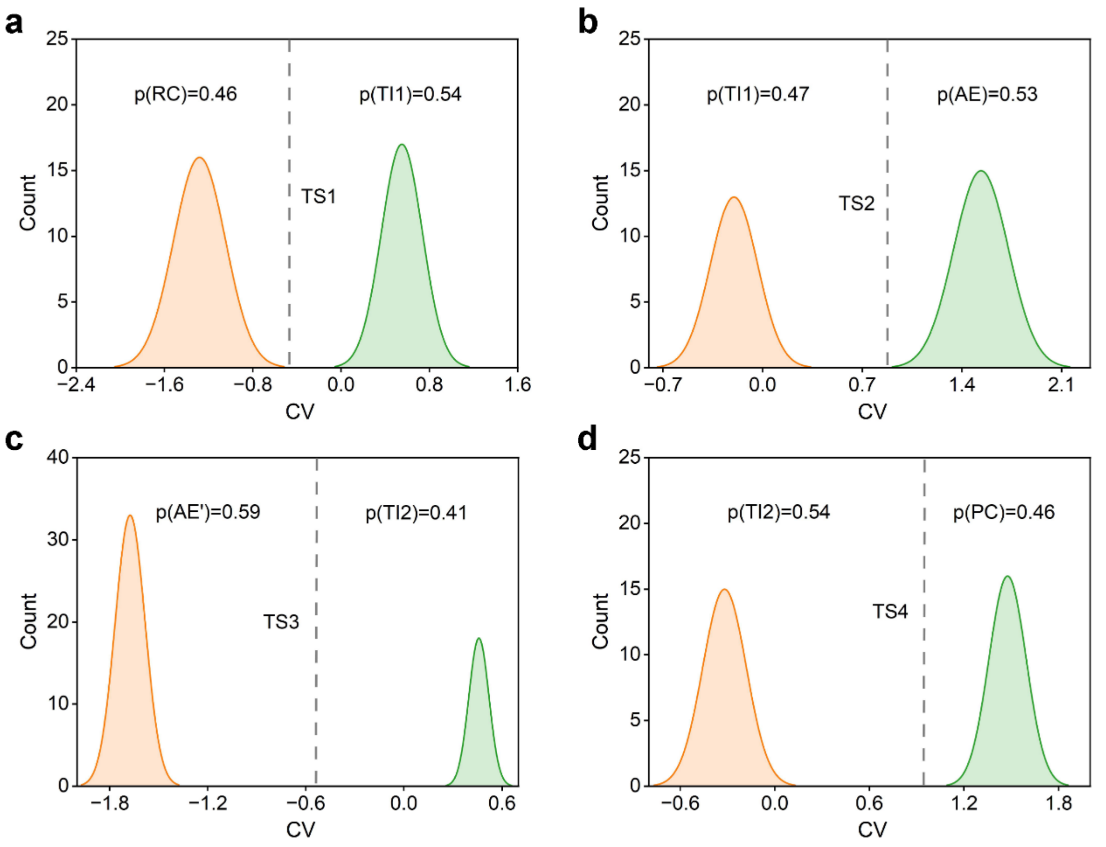

Figure S7. Committor probability analysis for the validation of the transition states. Distributions of the final states at the end of unbiased simulations of the TS ensembles for step i (a), step ii (b), step iii (c), and step iv (d). The committor probability was estimated as the ratio between number of trajectories reaching each endpoint to the total number of simulations.

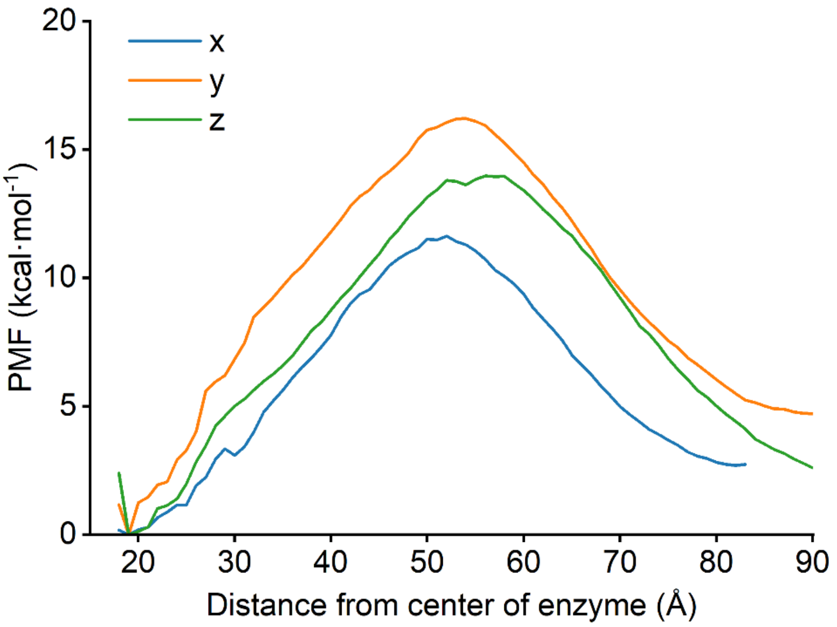

Figure S8. Free energy profiles of $MHET_2$ release from the active site of the LCC^{ICCG} variant along the x, y, and z axes. Free energies were calculated at the MM level.

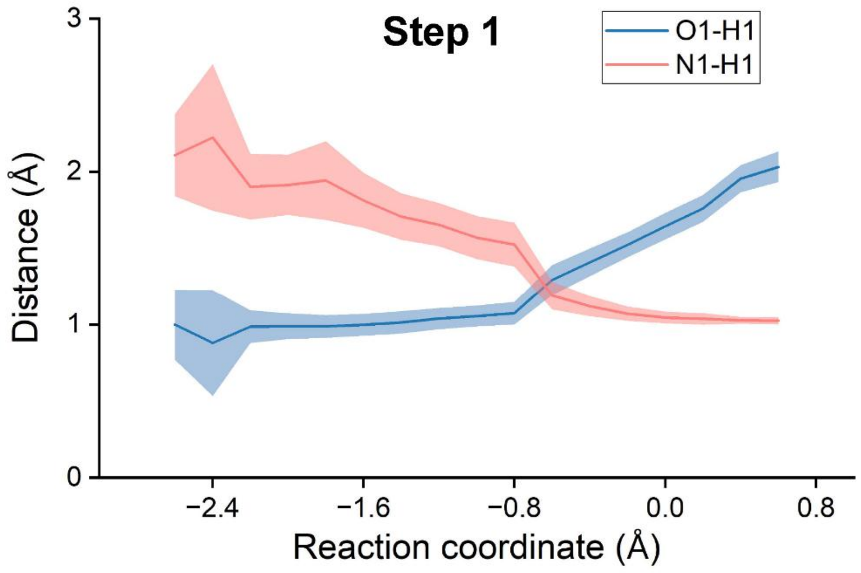

Figure S9. Evolution of the key distances O1-H1 and N1-H1 along the reaction coordinate of step i. Average values and standard deviation are calculated based on the last 15 ps QM/MM umbrella sampling MD.

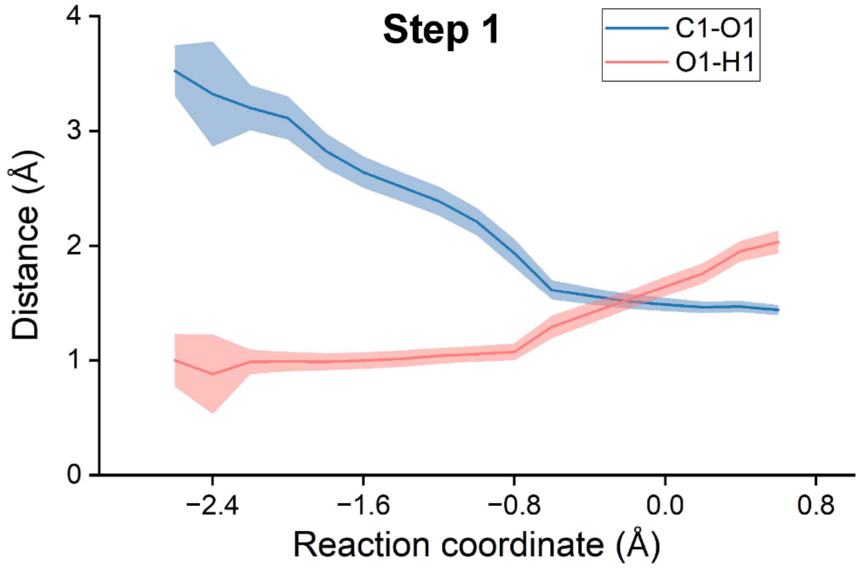

Figure S10. Evolution of the key distances O1-H1 (d_1) and C1-O1 (d_2) along the reaction coordinate defined by the CV_1. Average values and standard deviation are calculated based on the last 15 ps QM/MM umbrella sampling MD.

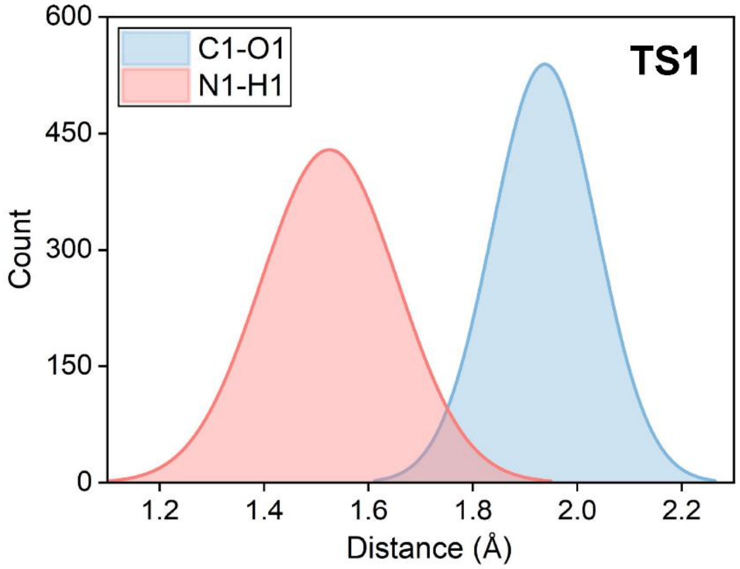

Figure S11. Distributions of C1-O1 and N1-H1 distances in TS1 of step i sampled by the last 15 ps QM/MM umbrella sampling MD. Distances are given in Å.

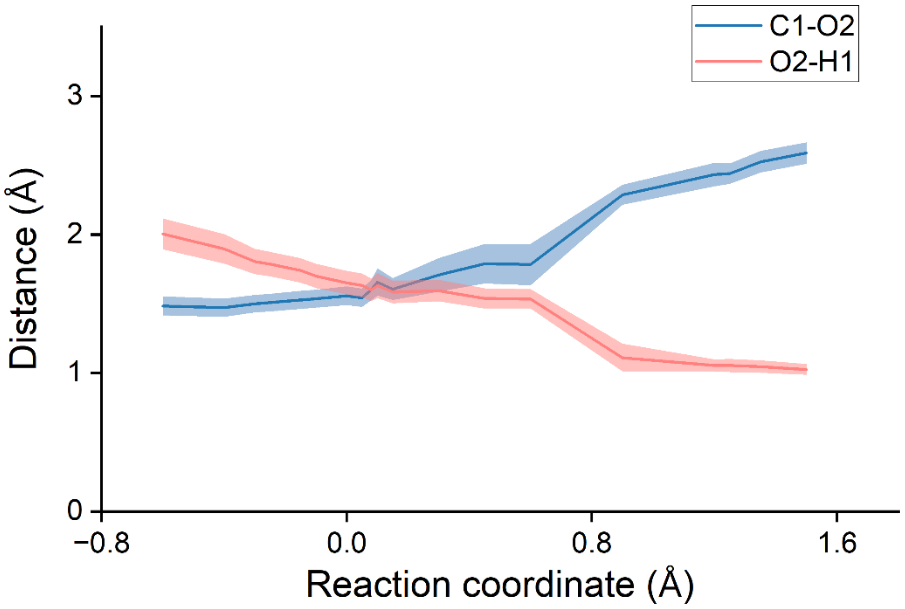

Figure S12. Evolution of the key distances C1-O2 (d_3) and O2-H1 (d_4) along the reaction coordinate defined by the CV_2. Average values and standard deviation are calculated based on the last 15 ps QM/MM umbrella sampling MD.

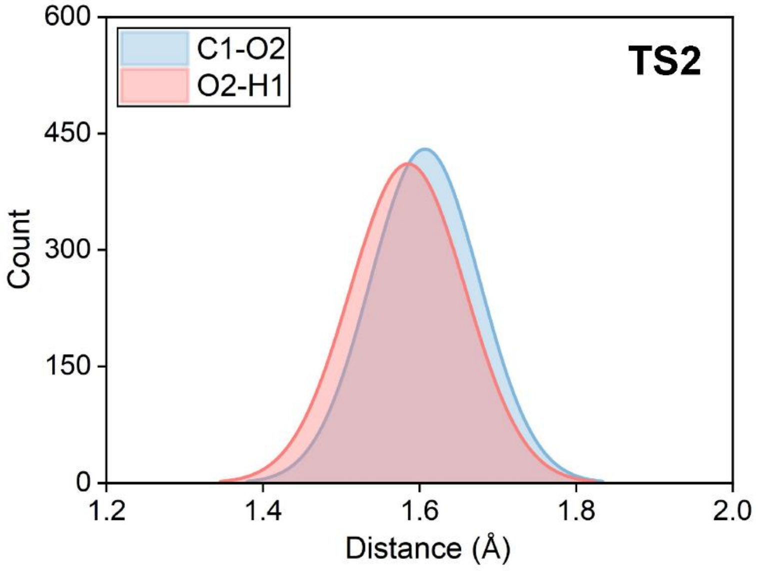

Figure S13. Distributions of C1-O2 and O2-H1 distances in a presentative structure of TS2 sampled by the last 15 ps QM/MM umbrella sampling MD. Distances are given in Å.

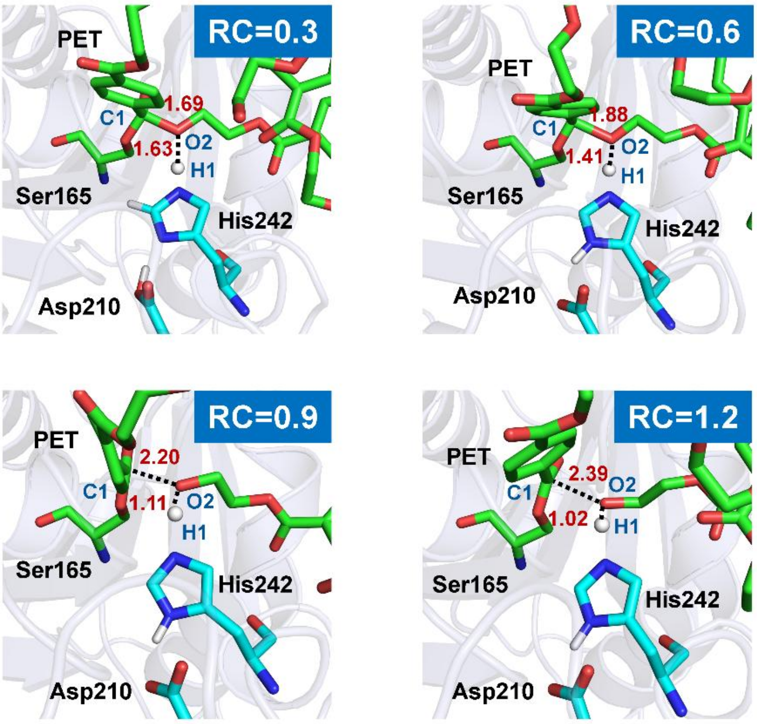

Figure S14. Representative structures of flat plateau transition state identified for step ii along the reaction coordination. Distances are given in Å.

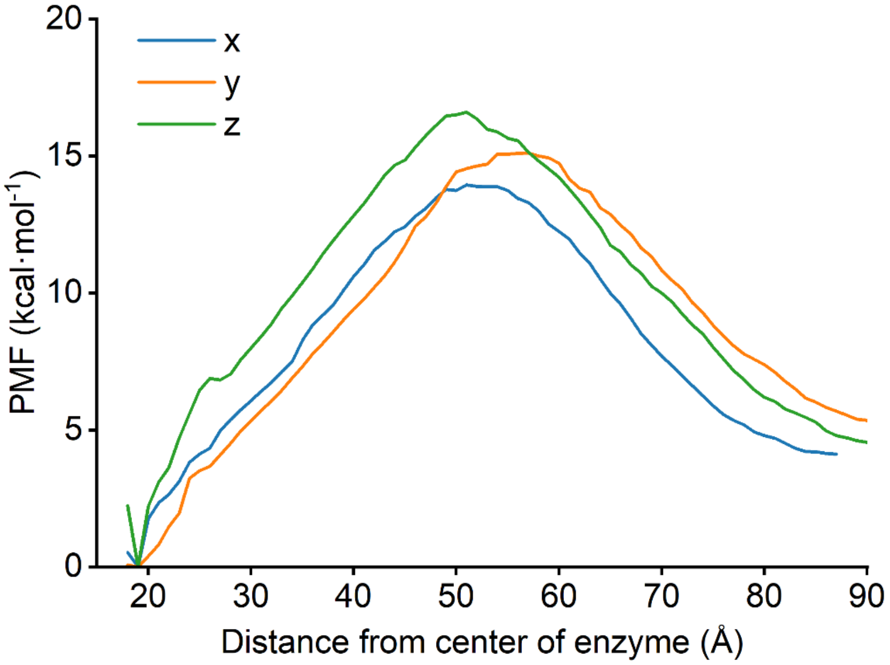

Figure S15. Free energy profiles of the departure of $MHET_4$ from the active site of the LCC^{ICCG} variant along the x, y, and z axes. Free energies were calculated at the MM level.

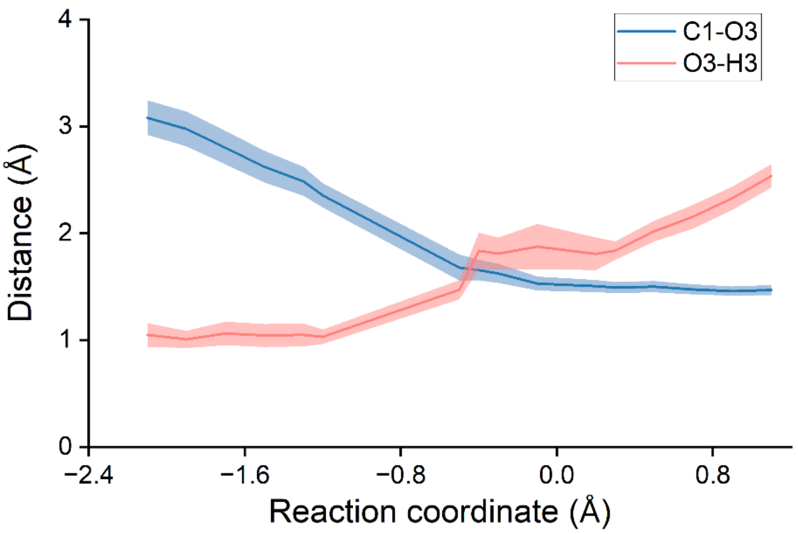

Figure S16 Evolution of the key distances O3-H3 (d_5) and C1-O3 (d_6) along the reaction coordinate defined by the CV_3. Average values and standard deviation are calculated based on the last 15 ps QM/MM umbrella sampling MD.

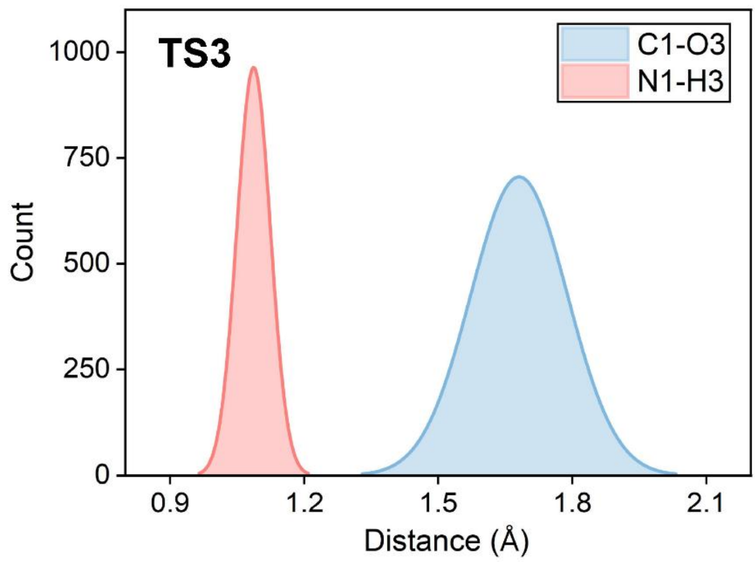

Figure S17. Distributions of C1-O3 and N1-H3 distances in TS3 of step iii sampled by the last 15 ps QM/MM umbrella sampling MD. Distances are given in Å.

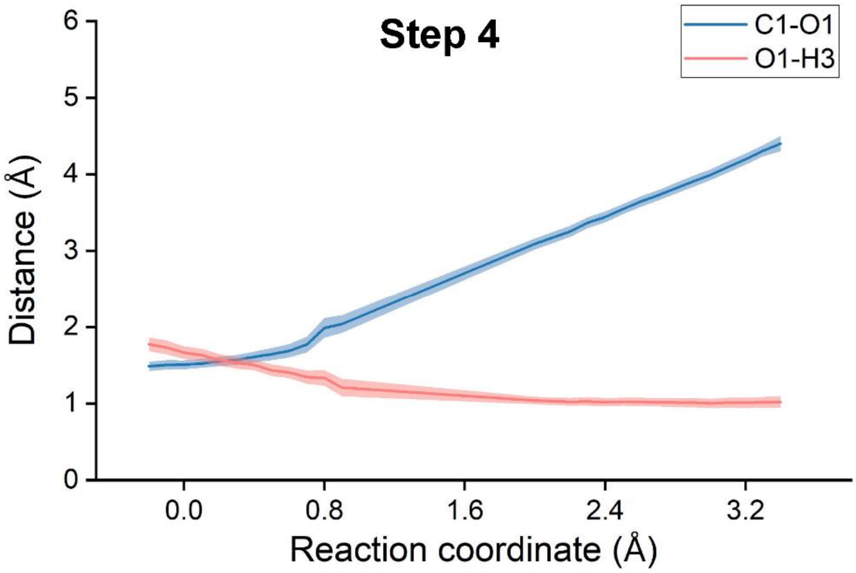

Figure S18. Evolution of the key distances C1-O1 (d_7) and O1-H3 (d_8) along the reaction coordinate defined by the CV_4. Average values and standard deviation are calculated based on the last 15 ps QM/MM umbrella sampling MD.

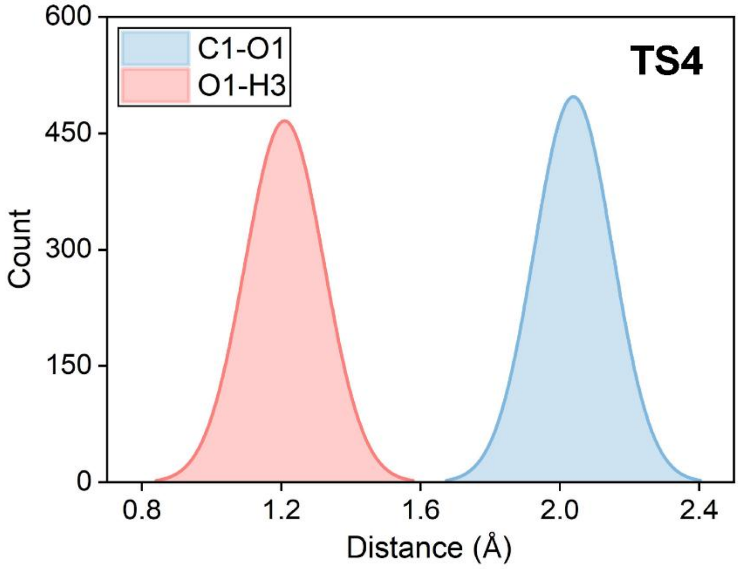

Figure S19. Distributions of C1-O1 and O1-H3 distances in TS4 of step iv sampled by the last 15 ps QM/MM umbrella sampling MD. Distances are given in Å.

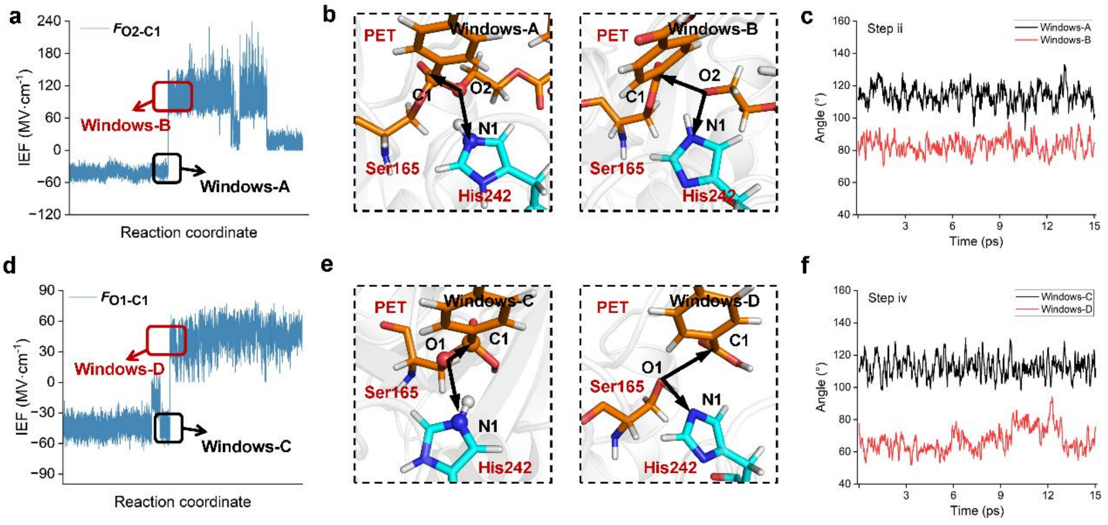

Figure S20. Molecular origin of field fluctuations. **a.** Fluctuation of calculated IEFs along the O2-C1 axis along the reaction coordinate in step ii. **b.** Angle C1-O2-N1 of representative structures in windows-A and windows-B, respectively. **c.** Fluctuations of angle C1-O2-N1 in windows-A and windows-B, respectively. **d.** Fluctuation of calculated IEFs along the O1-C1 axis along the reaction coordinate in step iv. **e.** Angle C1-O1-N1 of representative structures in windows-C and windows-D, respectively. **f.** Fluctuations of angle C1-O1-N1 in windows-C and windows-D, respectively.

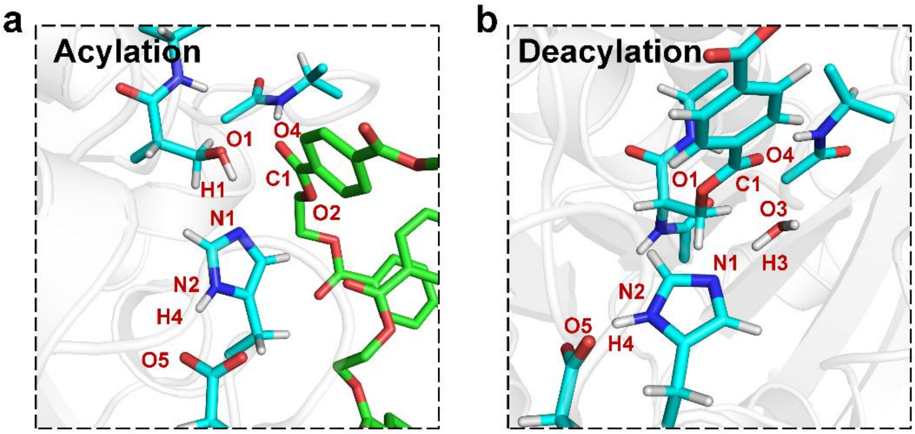

Figure S21. Labels of important atoms. **a.** Acylation stage. **b.** Deacylation stage.

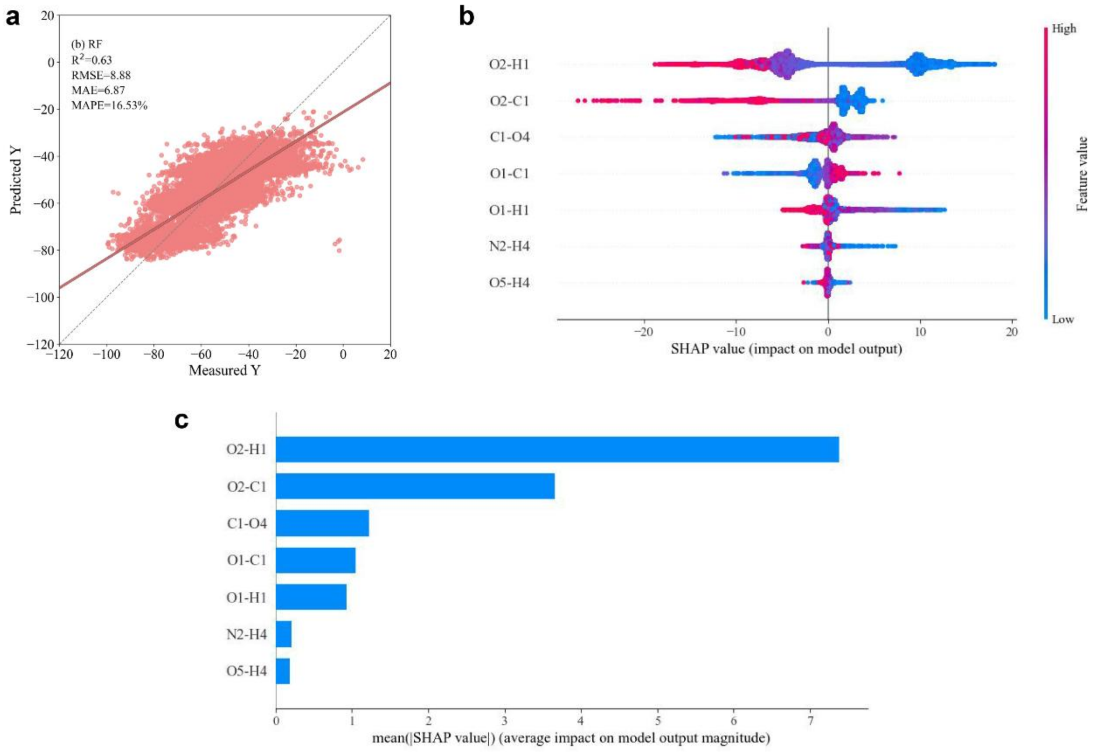

Figure S22. Analysis of IEFs along different directions in acylation stage by RF. **a.** Correlation of calculated results with predicted values. **b** and **c**. SHAP plot used to interpret the models.

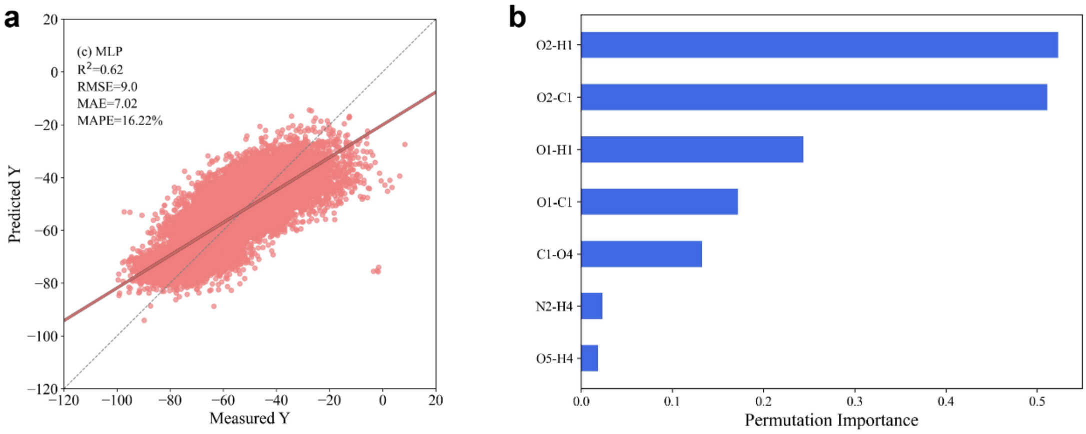

Figure S23. Analysis of IEFs along different directions in acylation stage by MLP. **a.** Correlation of calculated results with predicted values. **b.** Importance analysis.

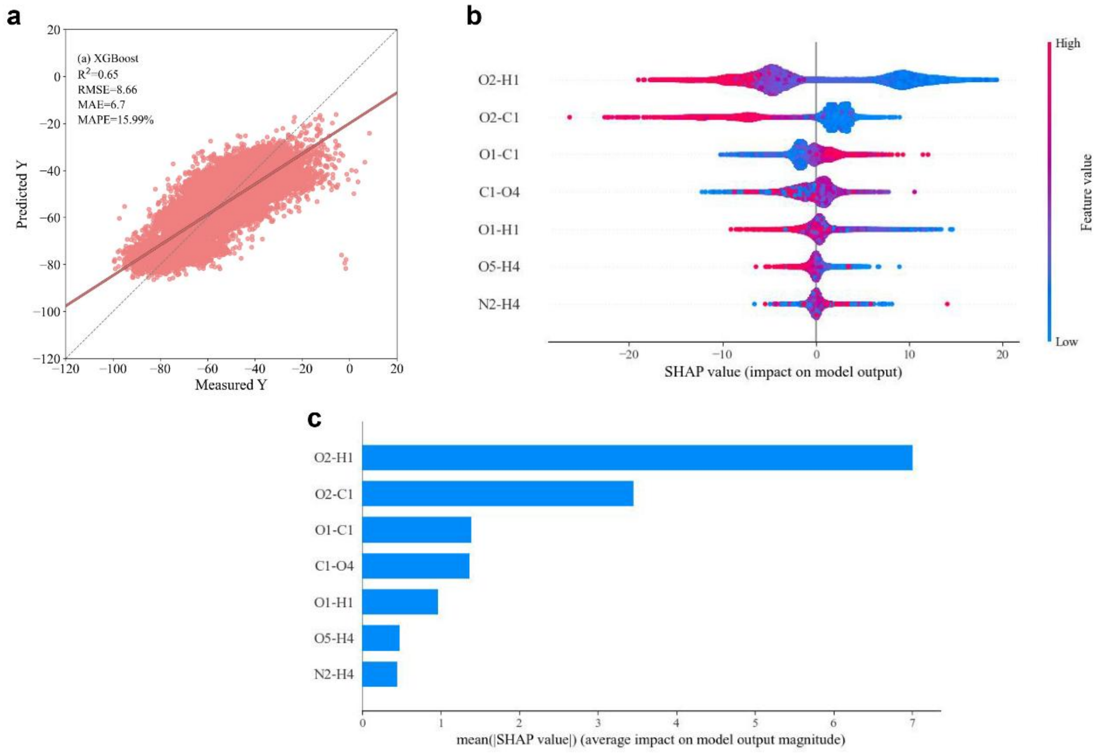

Figure S24. Analysis of IEFs along different directions in acylation stage by XGBoost. **a.** Correlation of calculated results with predicted values. **b** and **c**. SHAP plot used to interpret the models.

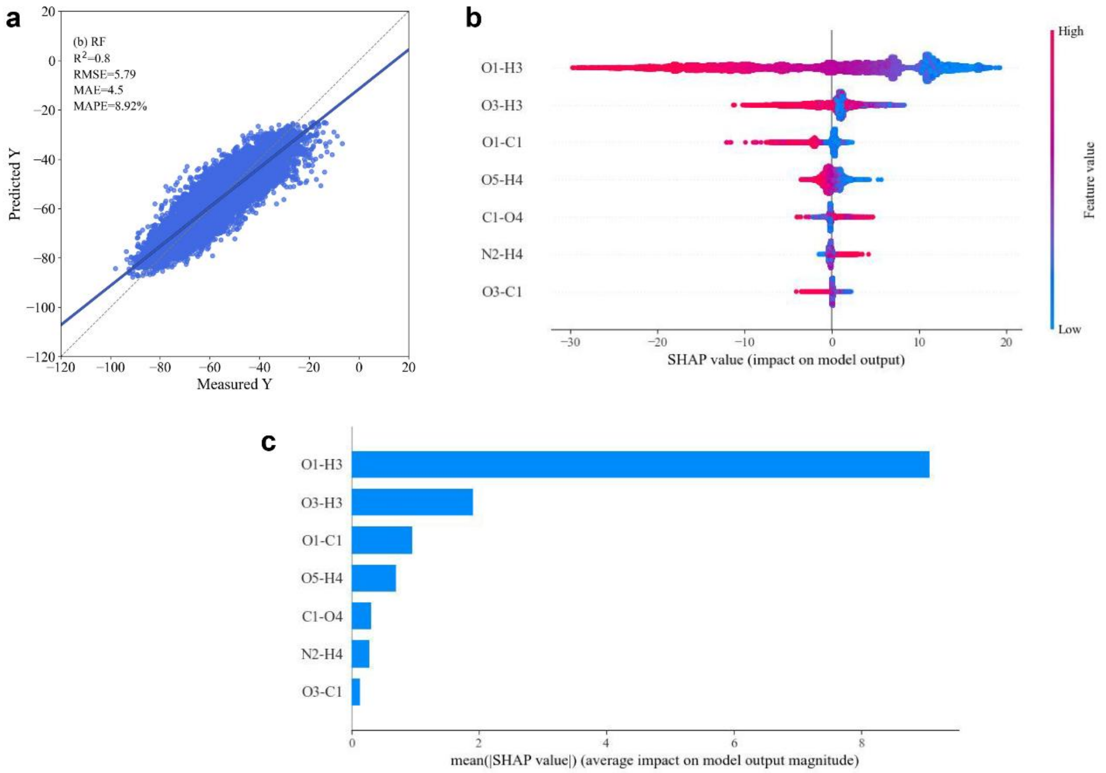

Figure S25. Analysis of IEFs along different directions in deacylation stage by RF. **a.** Correlation of calculated results with predicted values. **b** and **c**. SHAP plot used to interpret the models.

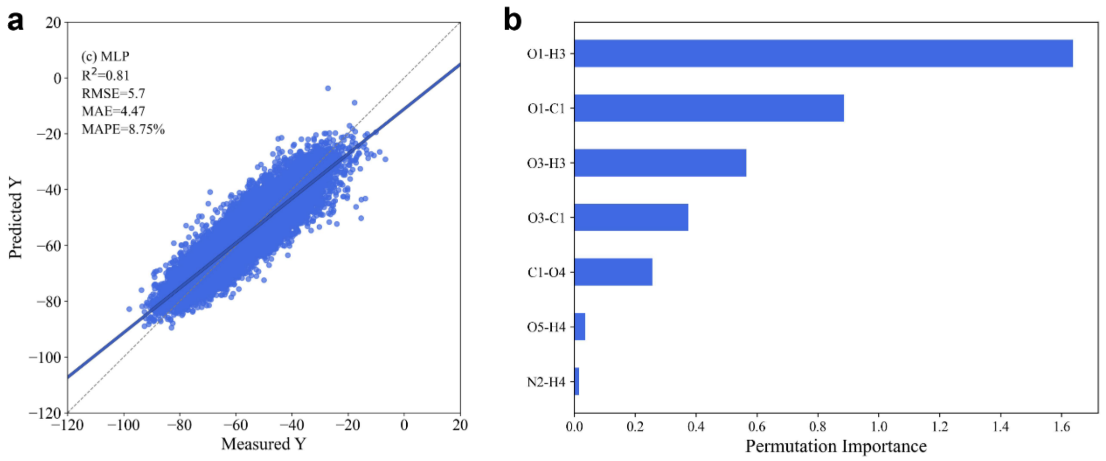

Figure S26. Analysis of IEFs along different directions in deacylation stage by MLP. **a.** Correlation of calculated results with predicted values. **b.** Importance analysis.

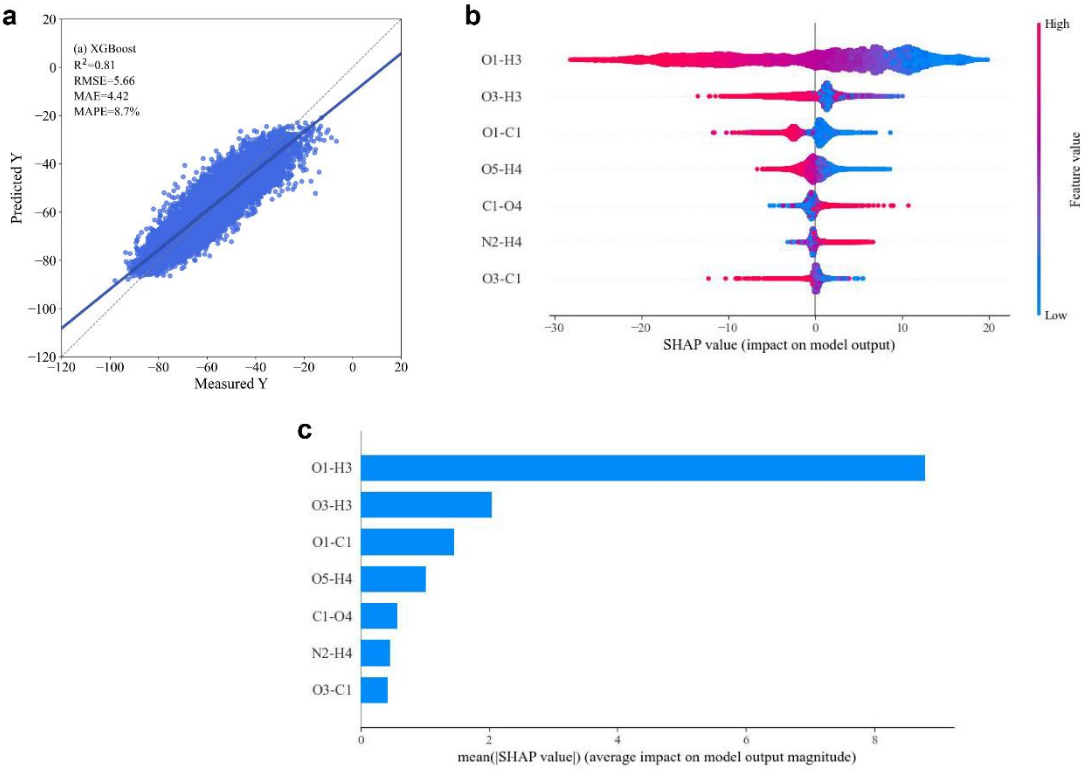

Figure S27. Analysis of IEFs along different directions in deacylation stage by XGBoost. **a.** Correlation of calculated results with predicted values. **b** and **c**. SHAP plot used to interpret the models.

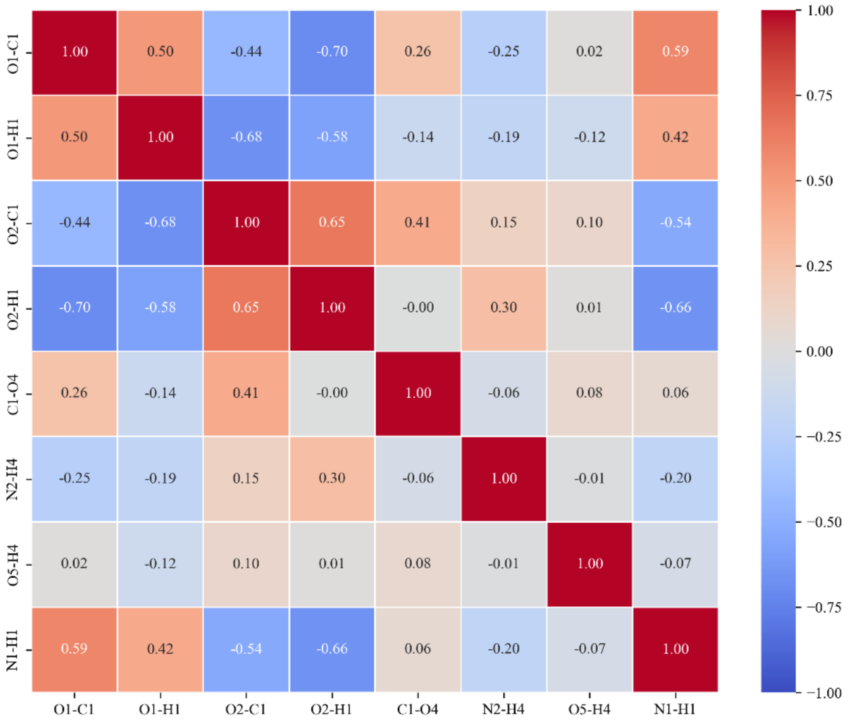

Figure S28. Correlation matrix between IEFs along different directions in acylation stage.

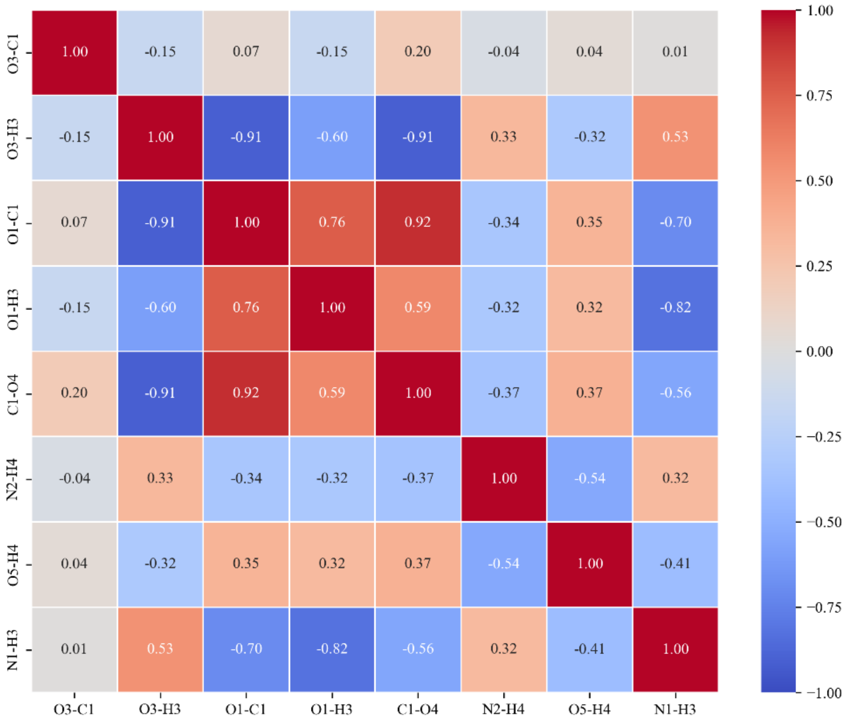

Figure S29. Correlation matrix between IEFs along different directions in deacylation stage.

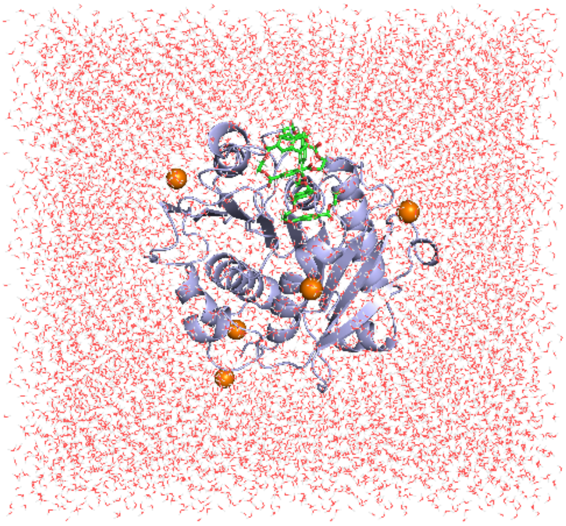

Figure S30. Depiction of the complete LCC^{ICCG}-PET model with the water box and chlorine counterions employed in classical MD simulations and QM/MM MD calculations.

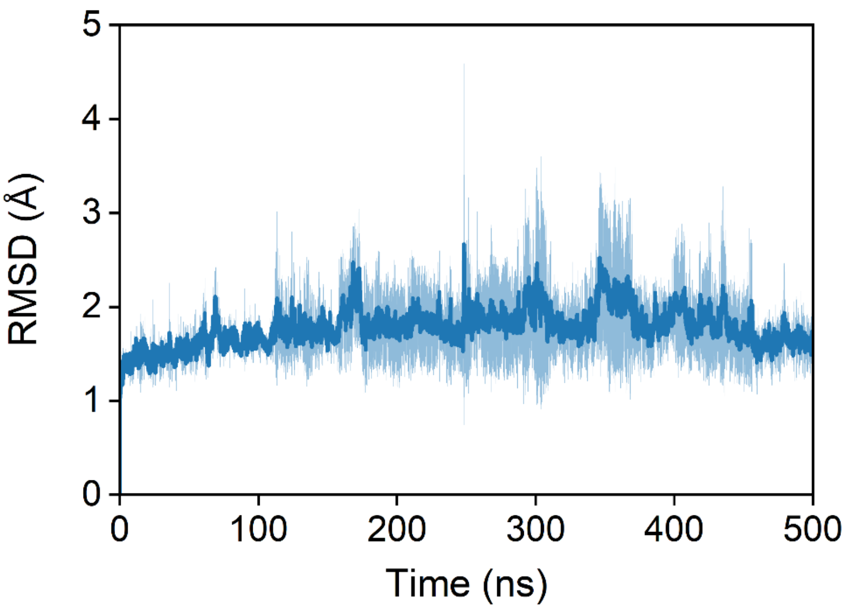

Figure S31. Calculated Root-Mean-Square Deviation (RMSD) for the backbone atoms of enzyme LCC^{ICCG} in complex with the substrate PET hexamer from three independent 500 ns MD simulations. The standard deviations are shown as semi-transparent shadings.

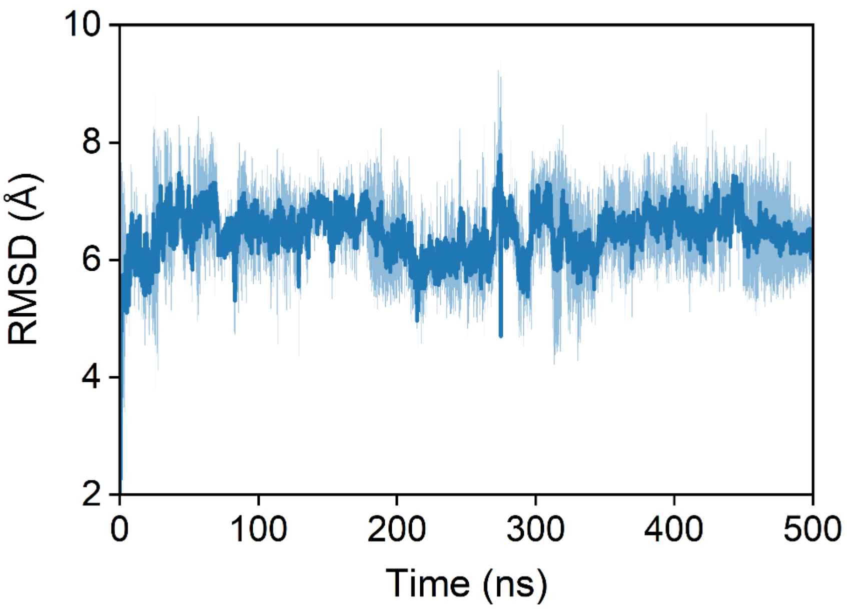

Figure S32. Calculated Root-Mean-Square Deviation (RMSD) of heavy atoms of the PET hexamer substrate within the active site of LCC^{ICCG} from three independent 500 ns MD simulations. The standard deviations are shown as semi-transparent shadings.

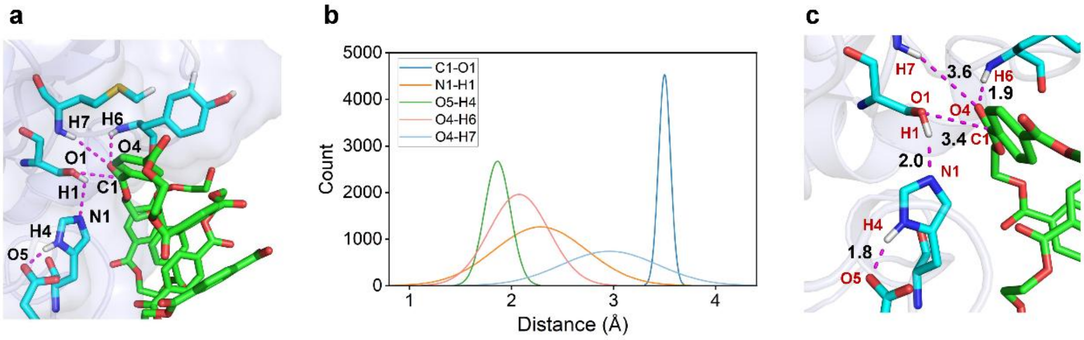

Figure S33. Representative conformation used in QM/MM calculations. **a.** Binding conformation of the model substrate PET hexamer in the active site of LCC^{ICCG}. The substrate and active-site residues are shown in green and cyan sticks, respectively. **b.** Distribution of key distances during the 500 ns MD simulation. Blue, orange, green, red, and light blue represent the C1-O1, N1-H1, O5-H4, O4-H6, and O4-H7 distances, respectively. **c.** Conformation extracted from the classical MD trajectory for QM/MM simulations.

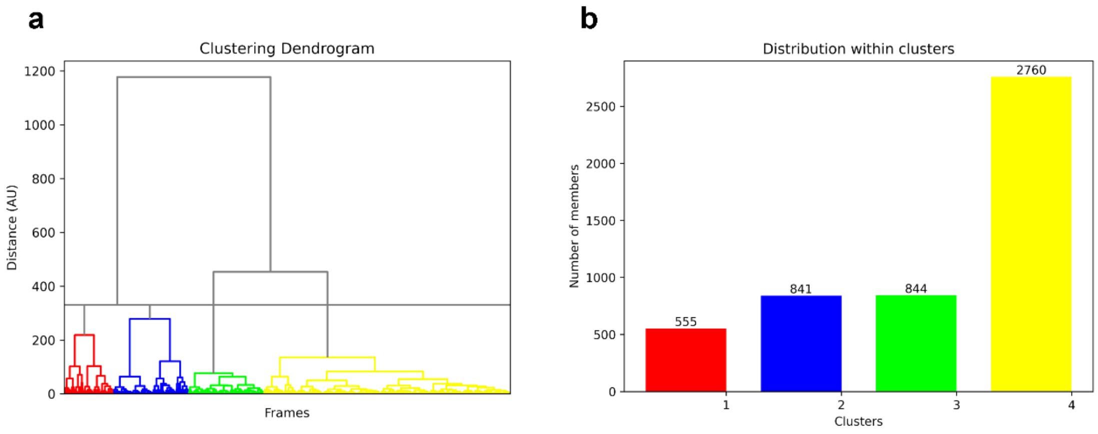

Figure S34. TTClust analysis of substrate conformations during the 500 ns MD simulation. **a.** Four clusters were identified. **b.** Barplot numbers of frames within clusters. The cluster color code is the same between the dendrogram and histogram. The heavy atoms of the PET hexamer were used for RMSD calculations.

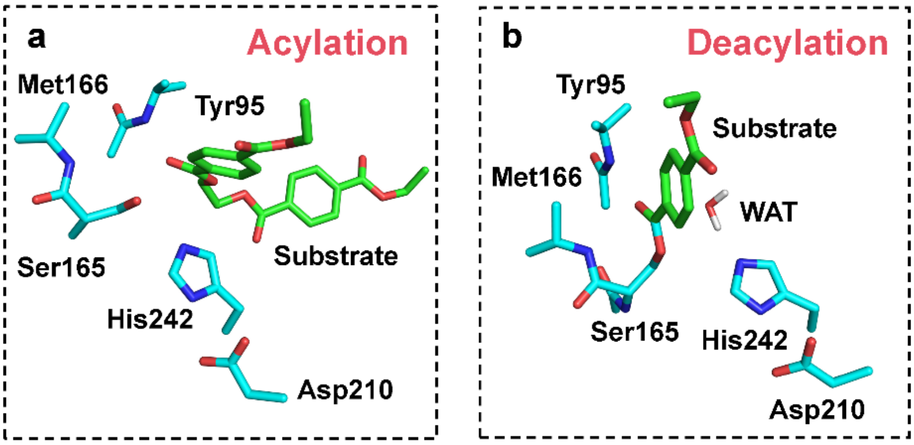

Figure S35. QM region using in the QM/MM calculations. **a.** QM region of the acylation stage. **b.** QM region of the deacylation stage.

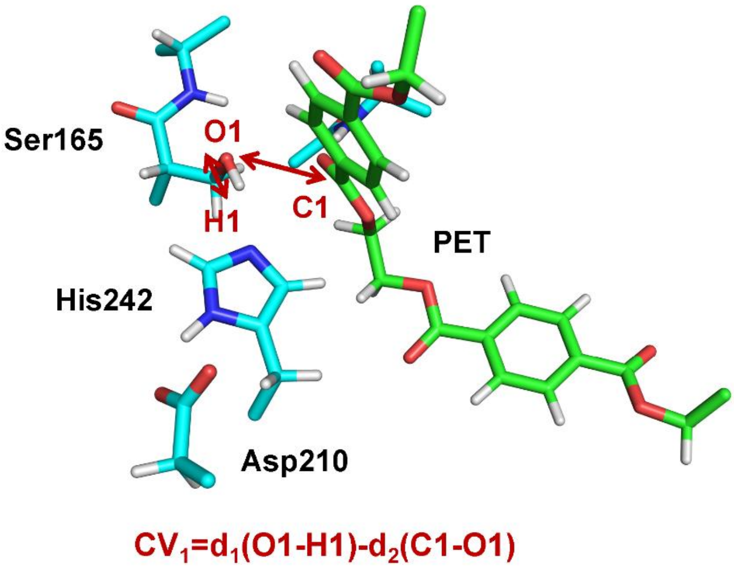

Figure S36. Defined collective variable for step i. Elongation of d_1 drives proton transfer, while shortening of d_2 drives nucleophilic attack.

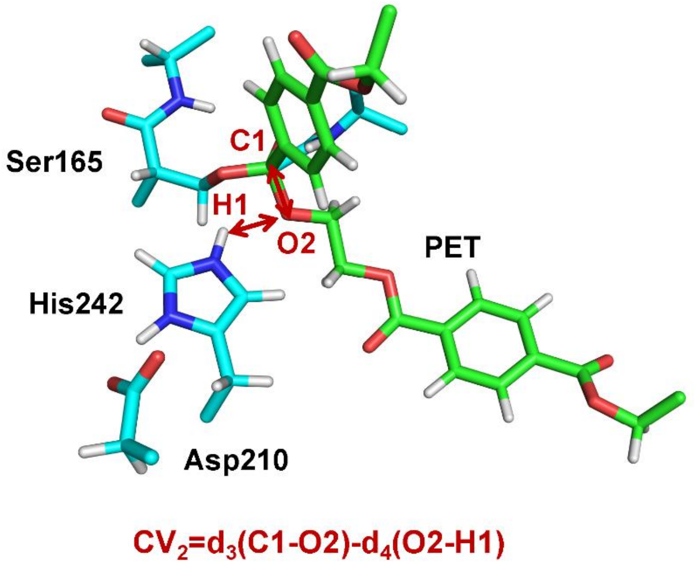

Figure S37. Defined collective variable for step ii. Elongation of d_3 drives cleavage of the ester bond, while shortening of d_4 drives proton transfer.

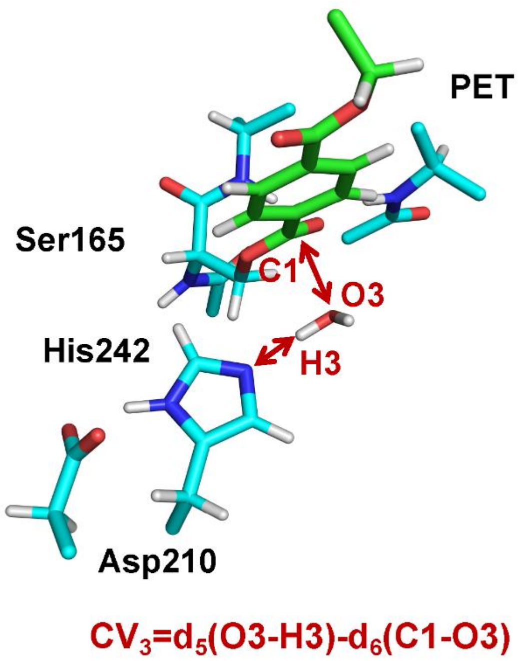

Figure S38. Defined collective variable for step iii. Elongation of d_5 drives proton transfer, while shortening of d_6 drives nucleophilic attack.

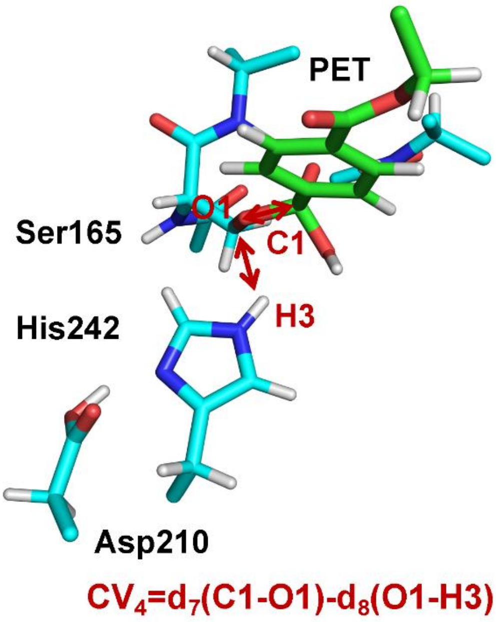

Figure S39. Defined collective variable for step iv. Elongation of d_7 drives cleavage of the C-O bond, while shortening of d_8 drives proton transfer.

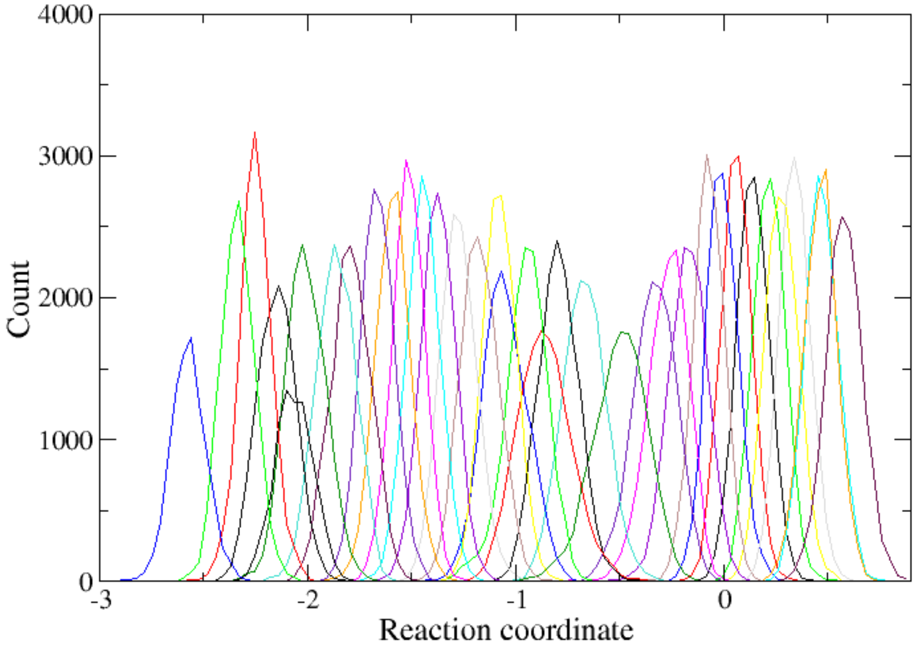

Figure S40. Overlap of the umbrella sampling window for the step i.

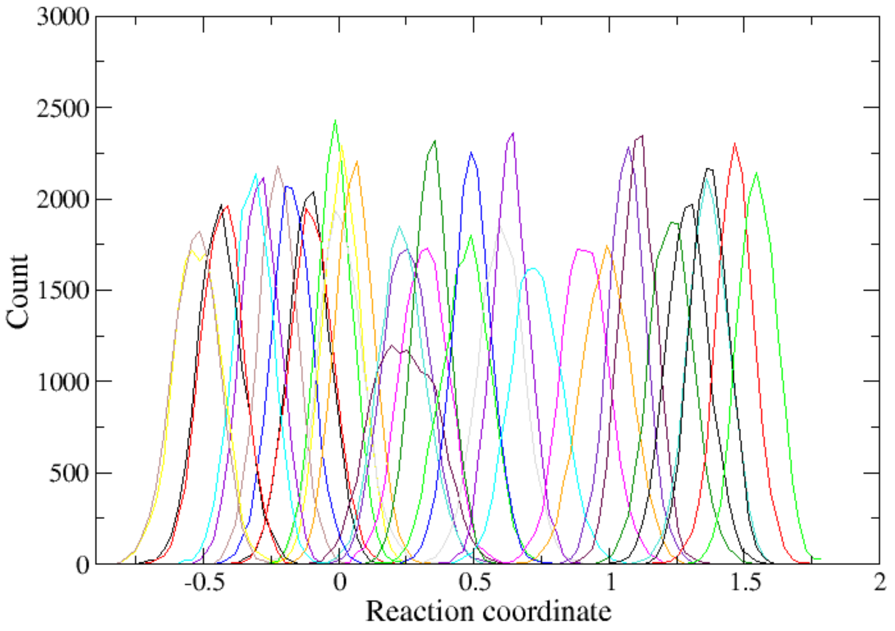

Figure S41. Overlap of the umbrella sampling window for the step ii.

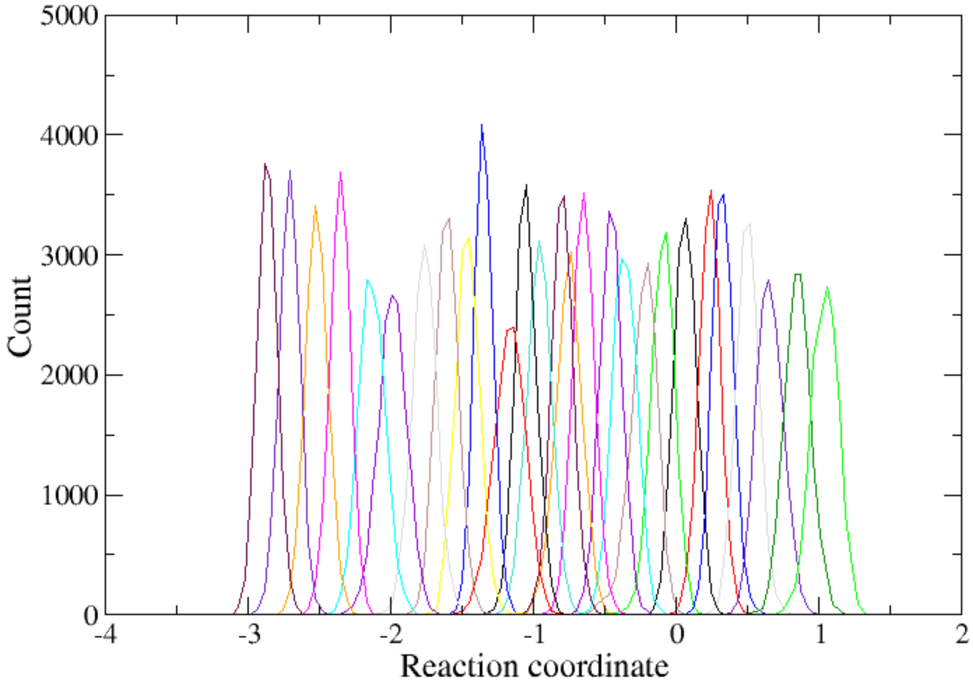

Figure S42. Overlap of the umbrella sampling window for the step iii.

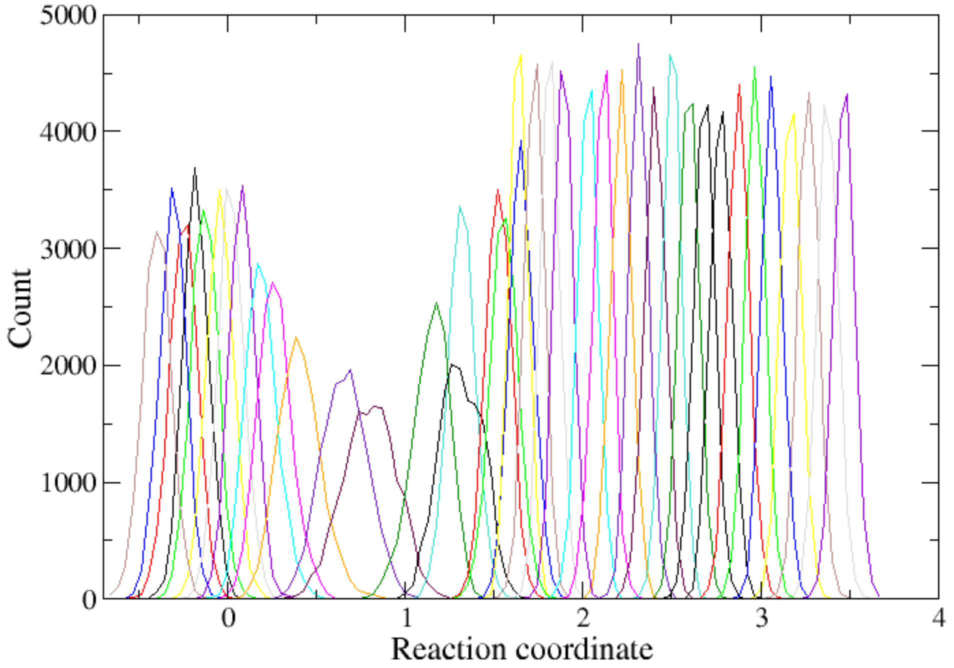

Figure S43. Overlap of the umbrella sampling window for the step iv.

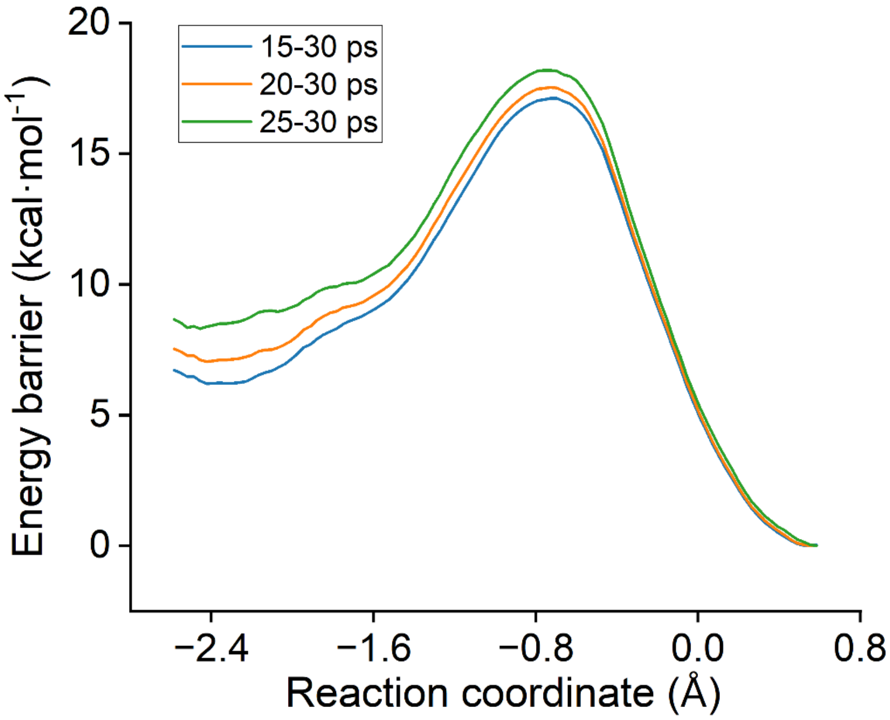

Figure S44. Free energy profiles of step i along the reaction coordinate obtained from different time blocks. Free energies were calculated at the M06-2X/6-31G(d)//MM level of theory.

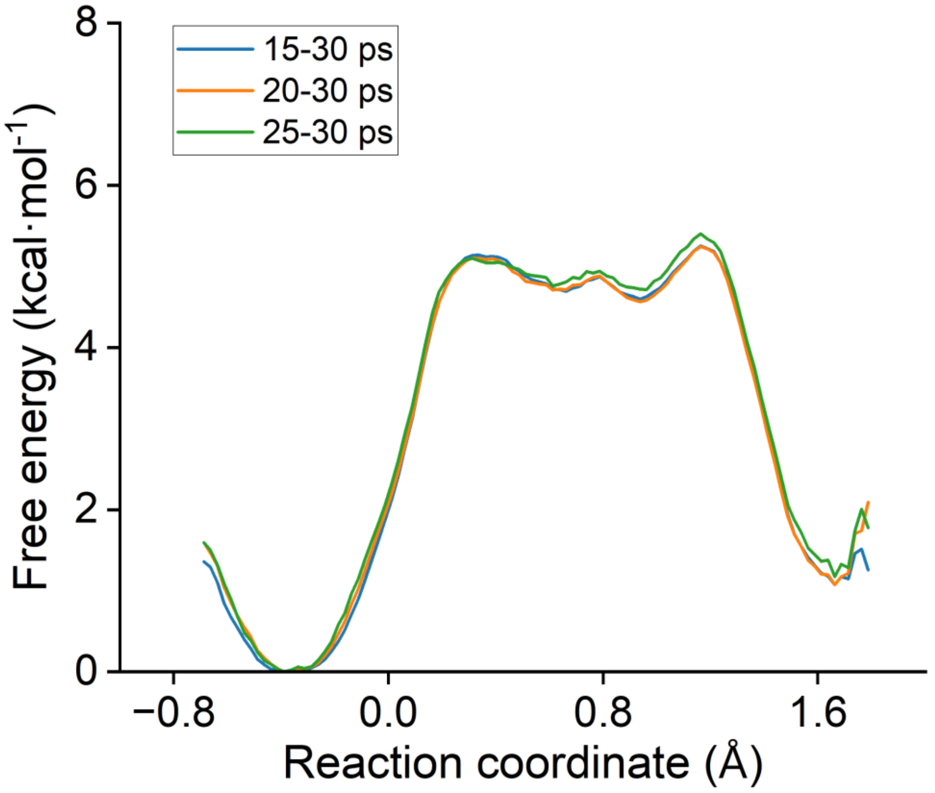

Figure S45. Free energy profiles of step ii along the reaction coordinate obtained from different time blocks. Free energies were calculated at the M06-2X/6-31G(d)//MM level of theory.

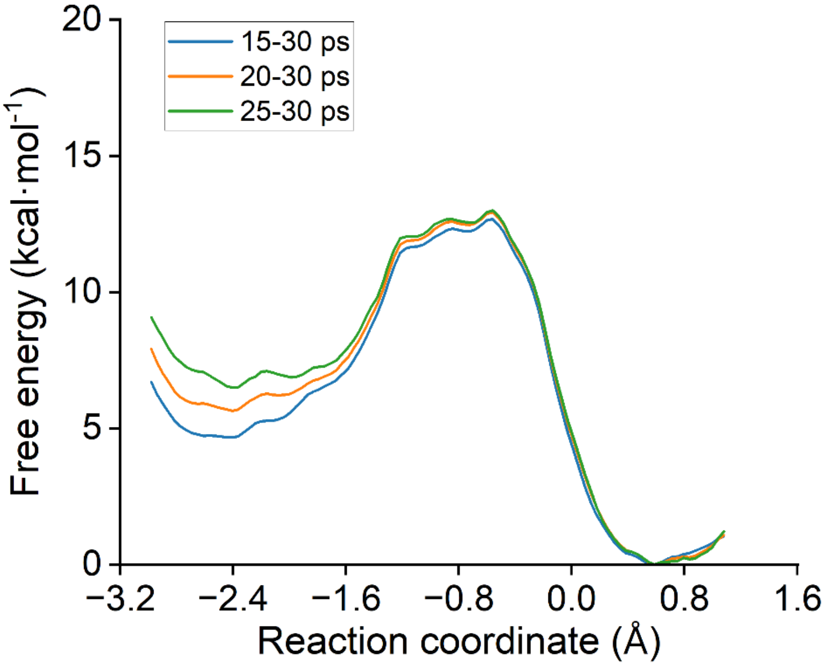

Figure S46. Free energy profiles of step iii along the reaction coordinate obtained from different time blocks. Free energies were calculated at the M06-2X/6-31G(d)//MM level of theory.

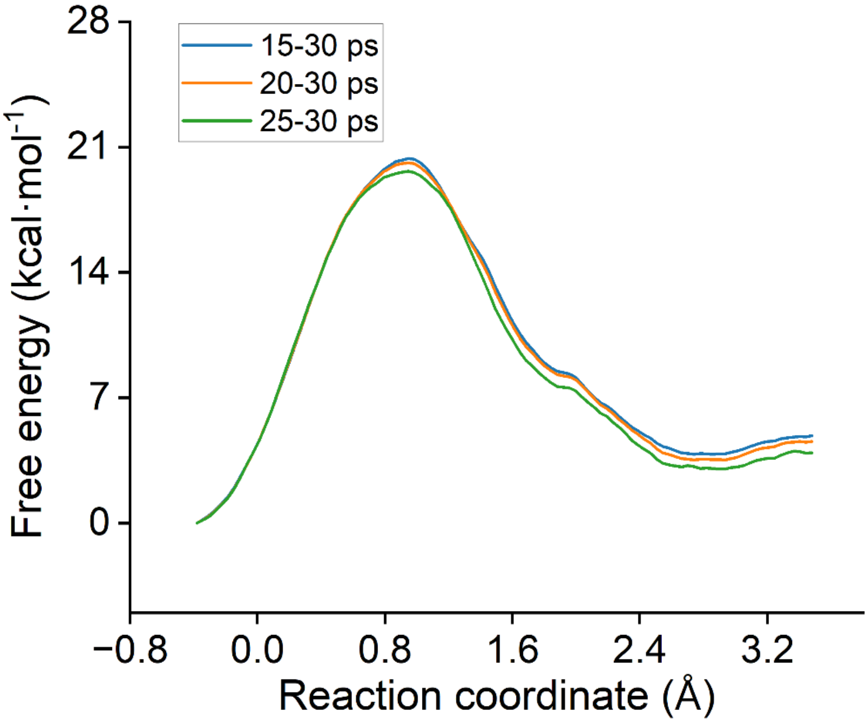

Figure S47. Free energy profiles of step iv along the reaction coordinate obtained from different time blocks. Free energies were calculated at the M06-2X/6-31G(d)//MM level of theory.

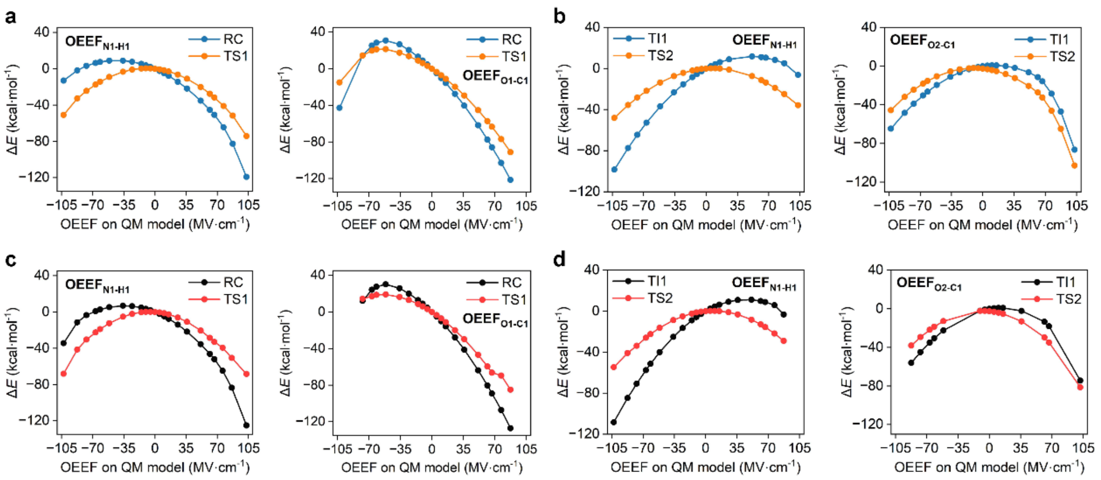

Figure S48. Effects of different levels of theory on OEEF calculations. **a.** Effects of OEEFs along the N1-H1 and O1-C1 axes on the energies of the reactant (RC) and transition state (TS1) in step i, calculated at the M06-2X/6-31G(d) level of theory. **b.** Effects of OEEFs along the N1-H1 and O2-C1 axes on the energies of the reactant (TI1) and transition state (TS2) in step ii, calculated at the M06-2X/6-31G(d) level of theory. **c.** Effects of OEEFs along the N1-H1 and O1-C1 axes on the energies of the reactant (RC) and transition state (TS1) in step i, calculated at the M06-2X/def2-TZVP level of theory. **d.** Effects of OEEFs along the N1-H1 and O2-C1 axes on the energies of the reactant (TI1) and transition state (TS2) in step ii, calculated at the M06-2X/def2-TZVP level of theory.

Table S1. pK_a values of titratable residues estimated by PROPKA3.0 software.

Residue ID	pK_a value	Residue ID	pK_a value
Asp53	3.25	Lys261	8.61
Asp98	2.95	ARG41	11.40
Asp126	2.95	ARG47	13.32
Asp129	1.70	ARG65	12.14
Asp155	2.71	ARG107	12.46
Asp193	2.88	ARG108	12.26
Asp210	2.45	ARG124	12.37
Asp265	6.11	ARG131	10.45
Asp267	3.27	ARG143	12.10
Asp279	2.67	ARG151	12.18
Asp284	3.75	ARG153	11.94
Glu176	3.66	ARG158	12.96
Glu208	3.92	ARG173	15.84
Glu236	4.92	ARG269	13.86
Lys182	10.06	ARG271	12.30
Lys194	9.27	ARG286	12.43
Lys232	9.00	ARG290	12.20

Table S2. The force constants of the harmonic biasing restraints applied to each window in step i.

Reaction coordinate/Å	Force constant/ kcal·mol^{-1}·Å^{-2}	Reaction coordinate/Å	Force constant/ kcal·mol^{-1}·Å^{-2}
-2.60	40	-0.85	60
-2.40	40	-0.80	40
-2.30	40	-0.75	60
-2.20	40	-0.70	40
-2.10	40	-0.65	60
-2.00	40	-0.60	40
-1.90	40	-0.50	40
-1.80	40	-0.40	40
-1.70	40	-0.30	40
-1.60	40	-0.20	40
-1.50	40	-0.10	40
-1.40	40	0.00	40
-1.30	40	0.10	40
-1.20	40	0.20	40
-1.10	40	0.30	40
-1.00	40	0.40	40
-0.95	60	0.50	40
-0.90	40	0.60	40

Table S3. The force constants of the harmonic biasing restraints applied to each window in step ii.

Reaction coordinate/Å	Force constant/ kcal·mol^{-1}·Å^{-2}	Reaction coordinate/Å	Force constant/ kcal·mol^{-1}·Å^{-2}
-0.60	40	0.34	50
-0.55	40	0.35	60
-0.45	40	0.45	40
-0.40	40	0.50	60
-0.30	40	0.60	40
-0.25	40	0.65	60
-0.15	40	0.75	40
-0.10	40	0.90	40
0.00	40	1.05	40
0.05	40	1.10	70
0.08	65	1.15	70
0.10	65	1.20	40
0.15	40	1.21	50
0.30	40	1.22	50
0.31	50	1.25	460
0.32	50	1.35	40
0.33	50	1.50	40

Table S4. The force constants of the harmonic biasing restraints applied to each window in step iii.

Reaction coordinate/Å	Force constant/ $kcal \cdot mol^{-1} \cdot Å^{-2}$	Reaction coordinate/Å	Force constant/ $kcal \cdot mol^{-1} \cdot Å^{-2}$
-2.90	60	-0.70	40
-2.70	60	-0.60	60
-2.50	60	-0.58	50
-2.30	60	-0.54	50
-2.10	40	-0.50	40
-1.90	40	-0.40	60
-1.70	40	-0.30	40
-1.50	40	-0.10	40
-1.30	40	0.20	60
-1.20	60	0.30	40
-1.10	65	0.50	40
-1.00	60	0.70	40
-0.90	40	0.90	40
-0.80	60	1.10	40

Table S5. The force constants of the harmonic biasing restraints applied to each window in step iv.

Reaction coordinate/Å	Force constant/ kcal·mol^{-1}·Å^{-2}	Reaction coordinate/Å	Force constant/ kcal·mol^{-1}·Å^{-2}
-0.30	40	1.60	100
-0.20	40	1.70	100
-0.10	40	1.80	100
0.00	40	1.90	100
0.10	40	2.00	100
0.20	40	2.10	100
0.30	40	2.20	100
0.40	40	2.30	100
0.50	40	2.40	100
0.60	40	2.50	100
0.70	40	2.60	100
0.80	40	2.70	100
0.90	40	2.80	100
1.00	40	3.00	100
1.10	40	3.10	100
1.20	40	3.20	100
1.30	40	3.30	100
1.40	40	3.40	100
1.50	40	3.50	100

Table S6. Statistical uncertainty of the free energy.

Elementary step	Statistical uncertainty/kcal·mol^{-1}
Step i	0.01-0.04
Step ii	0.02-0.06
Step iii	0.01-0.05
Step iv	0.01-0.05

Table S7. The windows numbers used for umbrella sampling of substrate binding, and the release of products $MHET_2$ and $MHET_4$.

	Axes	Windows numbers
	x	73
Substrate binding	y	73
	z	73
	x	66
Release of $MHET_2$	y	77
	z	78
	x	71
Release of $MHET_4$	y	76
	z	77

Table S8. Seven activity-related internal electric field features for acylation and deacylation stages, respectively.

Acylation stage	Deacylation stage
$F_{\text{O1-C1}}$	$F_{\text{O1-C1}}$
$F_{\text{O1-H1}}$	$F_{\text{O3-H3}}$
$F_{\text{O2-C1}}$	$F_{\text{O3-C1}}$
$F_{\text{O2-H1}}$	$F_{\text{O1-H3}}$
$F_{\text{C1-O4}}$	$F_{\text{C1-O4}}$
$F_{\text{O5-H4}}$	$F_{\text{O5-H4}}$
$F_{\text{N2-H4}}$	$F_{\text{N2-H4}}$

The Cartesian coordinates of the QM atoms for the initial structure

C 42.129514 48.091410 39.161798
C 43.115816 49.083134 39.613473
O 44.296685 48.785391 39.822183
N 42.672559 50.339472 39.790766
H 41.687960 50.559046 39.637856
C 43.571064 51.455186 40.040919
H 44.518987 51.151543 39.575413
C 43.055418 52.697645 39.375440
C 43.845331 51.647401 41.535029
C 38.296442 47.283733 36.131010
C 38.302954 48.795705 35.861383
H 37.363580 49.110893 35.399708
C 38.375117 49.536355 37.189825
H 37.676587 49.017262 37.855232
H 39.372975 49.412073 37.649929
O 38.034287 50.896371 37.089531
H 37.345446 51.047533 37.775246
C 39.368540 49.129182 34.815919
O 39.253465 48.722769 33.654679
N 40.518997 49.736384 35.203252
H 40.568657 50.164556 36.119668
C 41.562635 50.017040 34.236678
H 41.032075 50.464744 33.383942
C 42.569287 50.993576 34.803024
C 42.251225 48.754043 33.698506
C 28.310498 51.432115 40.854439
C 29.044985 50.242402 40.212645
H 29.282015 49.462480 40.944593
H 28.459180 49.735165 39.432710
C 30.407118 50.561121 39.531302
O 30.857726 51.719676 39.537644
O 30.963662 49.541999 39.024727
C 32.563871 48.832650 41.641200
C 32.964225 50.308001 41.416045
H 32.100304 50.915679 41.142688
H 33.397798 50.757507 42.321398
C 33.991178 50.476360 40.344360
N 33.687843 50.157801 39.044459
H 32.710113 49.926281 38.770938
C 34.816123 50.273490 38.315469
H 34.858782 50.069077 37.255631
N 35.844325 50.664155 39.054536
C 35.332604 50.788279 40.329084
H 35.943446 51.105643 41.163794
C 39.442034 50.312325 41.953959
O 38.784219 51.478624 41.445629
C 39.098154 51.845020 40.192794
O 39.902649 51.238154 39.513857
C 38.441609 53.113185 39.764480
C 37.691334 53.889764 40.652878
C 37.258776 55.154016 40.267130
C 37.534389 55.618705 38.979721
C 38.210679 54.805411 38.064689
C 38.684252 53.561282 38.461472
C 37.101418 56.971292 38.519218
O 37.002627 57.259745 37.349178
O 36.864297 57.824146 39.524788
C 36.274884 59.098755 39.180146
C 37.246021 60.086441 38.573738

C 38.589816 49.705249 43.044960
O 38.752129 50.445951 44.259393
C 37.754277 50.250170 45.153201
O 36.785474 49.579612 44.909434
C 37.960896 50.951381 46.455977
C 36.843089 51.090785 47.282177
C 36.968324 51.691656 48.527979
C 38.222480 52.125944 48.963309
C 39.337841 51.994748 48.132557
C 39.206986 51.420439 46.874664
C 38.432568 52.677207 50.336744
O 39.528012 52.836192 50.825479
O 37.278714 52.969349 50.956295
C 37.368307 53.416635 52.326802
C 37.735807 54.875959 52.434969
H 39.582742 49.594816 41.141906
H 40.432596 50.595093 42.330523
H 37.444613 53.507126 41.634044
H 36.702634 55.776238 40.957956
H 38.367055 55.166256 37.054652
H 39.208096 52.910152 37.771908
H 35.458922 58.897598 38.481372
H 35.875933 59.438102 40.140041
H 37.538163 49.715080 42.747686
H 38.900256 48.670973 43.227803
H 35.888482 50.716510 46.931953
H 36.108518 51.802503 49.178360
H 40.298805 52.333534 48.502471
H 40.067618 51.304904 46.225553
H 36.373812 53.189437 52.720608
H 38.116242 52.795888 52.825159

The Cartesian coordinates of the QM atoms for the representative TSs of each elementary step.

TS1

C 42.172661 48.208658 39.075395
C 43.138947 49.189885 39.600394
O 44.329618 48.903046 39.777356
N 42.656435 50.413616 39.865542
H 41.684080 50.627364 39.604680
C 43.510870 51.572327 40.090603
H 44.445789 51.342566 39.554802
C 42.861235 52.795362 39.517161
C 43.865969 51.764370 41.566211
C 38.306550 47.446734 36.304124
C 38.362036 48.978103 36.169395
H 37.416912 49.365893 35.772483
C 38.542558 49.532880 37.603578
H 38.027834 48.789247 38.220086
H 39.597684 49.504927 37.910326
O 38.019349 50.806632 37.921827
H 36.964976 50.633690 38.497255
C 39.409621 49.268579 35.073415
O 39.234471 48.830545 33.930143
N 40.613375 49.814189 35.385543
H 40.761223 50.235825 36.297247
C 41.596166 50.022369 34.332488
H 41.017398 50.467141 33.509805
C 42.673048 50.971987 34.802716
C 42.209074 48.732286 33.757858
C 28.575543 51.409389 40.692404
C 29.200911 50.129816 40.119876
H 29.365109 49.363036 40.885117

H 28.581776 49.636793 39.357812
C 30.578089 50.303498 39.428116
O 31.125132 51.414791 39.354748
O 31.064321 49.217642 38.975584
C 32.529751 48.691938 41.667919
C 32.976390 50.146145 41.418658
H 32.136328 50.781699 41.142371
H 33.437466 50.599185 42.309754
C 33.999553 50.247188 40.329403
N 33.690462 49.868363 39.044328
H 32.702199 49.594463 38.786766
C 34.794894 49.955940 38.299793
H 34.853393 49.713816 37.252696
N 35.821638 50.379308 39.029032
C 35.337401 50.556719 40.307042
H 35.962782 50.918971 41.105755
C 39.303217 51.189437 41.488222
O 38.514505 51.047665 40.307208
C 39.081080 51.624067 39.142033
O 40.257885 51.341832 38.848815
C 38.613962 53.057027 38.919797
C 37.927795 53.777610 39.899626
C 37.667415 55.139752 39.724653
C 38.032095 55.774821 38.537331
C 38.676878 55.037694 37.533450
C 38.988898 53.701505 37.735643
C 37.657297 57.188922 38.227804
O 37.597245 57.600763 37.088944
O 37.390783 57.952496 39.295859
C 36.701195 59.210592 39.055093
C 37.559302 60.325239 38.502486
C 38.514512 50.510063 42.611871
O 38.897517 51.047957 43.893670
C 38.045529 50.689505 44.889929
O 37.137882 49.917057 44.713673
C 38.265081 51.392360 46.201326
C 37.163555 51.451719 47.062096
C 37.251775 52.111947 48.283238
C 38.464476 52.674579 48.688797
C 39.574190 52.594049 47.840955
C 39.469705 51.981238 46.594107
C 38.624084 53.290192 50.051220
O 39.671674 53.321022 50.654070
O 37.468623 53.788991 50.525894
C 37.464006 54.242091 51.897877
C 37.892854 55.684587 52.048979
H 40.292219 50.740987 41.350913
H 39.441395 52.254713 41.727054
H 37.584140 53.273756 40.797277
H 37.154345 55.703245 40.496053
H 38.920446 55.527825 36.597570
H 39.503372 53.127488 36.973379
H 35.883397 58.988892 38.363865
H 36.299320 59.438605 40.045883
H 37.449293 50.699444 42.471690
H 38.673465 49.428569 42.622375
H 36.236787 50.981966 46.757172
H 36.388364 52.170543 48.935760
H 40.515397 53.017126 48.174516
H 40.328940 51.936977 45.933698
H 36.429489 54.069028 52.206671
H 38.128640 53.580595 52.457930

TS2

C	42.269134	48.096187	38.936839
C	43.229958	49.094553	39.443854
O	44.393122	48.791599	39.740347
N	42.783288	50.352471	39.561588
H	41.843113	50.582525	39.229735
C	43.657511	51.490645	39.831291
H	44.613622	51.240939	39.344945
C	43.061298	52.720504	39.208211
C	43.939161	51.657539	41.323496
C	38.283922	47.226736	36.133215
C	38.346249	48.749831	35.925117
H	37.418649	49.104806	35.464648
C	38.461559	49.391740	37.299084
H	37.633682	49.016504	37.907123
H	39.385343	49.100814	37.804741
O	38.358664	50.817079	37.274264
H	37.012468	50.820197	39.776082
C	39.437981	49.086395	34.902787
O	39.312993	48.692783	33.736218
N	40.592508	49.671503	35.299220
H	40.694969	50.044409	36.243876
C	41.632548	49.910306	34.311780
H	41.102224	50.381401	33.470237
C	42.693988	50.839639	34.852207
C	42.248138	48.618679	33.747414
C	28.752330	51.355867	40.654954
C	29.390705	50.123465	39.978728
H	29.596403	49.315629	40.693356
H	28.750843	49.659425	39.214738
C	30.747787	50.363509	39.243013

O	31.266067	51.494691	39.200428
O	31.269691	49.317331	38.746440
C	32.344088	48.733646	41.713416
C	32.673304	50.228987	41.560416
H	31.812671	50.805326	41.224732
H	32.984760	50.678686	42.516306
C	33.822075	50.472475	40.636028
N	33.815474	50.158499	39.293996
H	32.922135	49.851866	38.813874
C	35.053642	50.304172	38.814003
H	35.353668	50.129209	37.791124
N	35.870941	50.701323	39.793151
C	35.113753	50.806992	40.935562
H	35.539585	51.077359	41.889952
C	39.201811	50.879809	40.941560
O	38.416785	50.896012	39.792121
C	39.285657	51.470327	38.018999
O	40.446175	51.055839	38.107600
C	38.988432	52.958081	38.073075
C	38.599157	53.654413	39.218924
C	38.407163	55.033339	39.176036
C	38.571708	55.730326	37.978837
C	38.962558	55.036728	36.829549
C	39.179420	53.665585	36.879658
C	38.230494	57.175671	37.839640
O	38.101344	57.712019	36.761568
O	38.053469	57.819845	39.002191
C	37.403440	59.114848	38.918402
C	38.313606	60.236715	38.479607
C	38.241259	50.931153	42.132900
O	38.853654	51.105731	43.432744

C 38.053318 50.726947 44.453817
O 37.066530 50.046548 44.296135
C 38.407376 51.306935 45.797807
C 37.402626 51.253958 46.769301
C 37.546795 51.918617 47.979888
C 38.731736 52.600592 48.263948
C 39.775447 52.579536 47.331795
C 39.607739 51.956879 46.096365
C 38.919647 53.331485 49.558796
O 39.991579 53.729146 49.958780
O 37.777497 53.506318 50.239525
C 37.886139 53.980531 51.608236
C 38.127827 55.466096 51.736041
H 39.836819 49.979004 41.003590
H 39.888007 51.749003 40.982816
H 38.411435 53.105034 40.130439
H 38.096016 55.568126 40.066113
H 39.072309 55.580163 35.898862
H 39.471953 53.133156 35.980508
H 36.577826 59.002176 38.211396
H 37.011034 59.243075 39.930743
H 37.565571 51.780677 41.990287
H 37.639627 50.022031 42.145583
H 36.497637 50.700923 46.557874
H 36.741290 51.914307 48.704900
H 40.708504 53.074724 47.577655
H 40.403399 51.981356 45.358930
H 36.935960 53.658922 52.041425
H 38.708810 53.420168 52.062688

TS3

C 32.805137 37.316794 26.160721
C 31.545096 37.199611 25.399322
O 30.419827 37.212517 25.911122
N 31.669653 37.083503 24.057664
H 32.605289 37.117368 23.633718
C 30.544246 37.384270 23.187172
H 30.094860 38.316018 23.574049
C 31.035240 37.564063 21.780822
C 29.463608 36.318065 23.279949
C 36.890786 36.966671 28.570308
C 36.519014 37.590526 27.279475
O 35.362477 37.906320 27.028558
N 37.530175 37.752656 26.394232
H 38.480259 37.512414 26.680745
C 37.251938 38.073698 24.999864
H 38.214792 38.199670 24.506779
C 36.527878 36.894810 24.351583
H 37.116712 35.990348 24.535881
H 35.540165 36.753539 24.790437
O 36.429766 37.086193 22.942077
C 35.171413 36.836494 22.438569
O 34.180652 37.272168 23.077018
C 35.122931 36.944753 20.926471
C 33.955908 36.499356 20.297052
C 33.733186 36.720405 18.948669
C 34.716162 37.362571 18.190579
C 35.915755 37.739930 18.792419
C 36.115089 37.547890 20.160216
H 37.033600 37.866582 20.634174
H 36.677900 38.194579 18.173304
C 34.544047 37.606765 16.733439
O 35.467615 37.730997 15.963925

O	33.249091	37.683178	16.363141
C	32.979634	37.739338	14.950026
C	33.240470	39.085558	14.318701
H	33.606396	36.984523	14.466234
H	31.925475	37.443847	14.896842
H	32.819359	36.385141	18.471552
H	33.238108	35.951016	20.891510
C	36.595513	39.458827	24.926989
O	37.281801	40.452762	25.197174
N	35.302615	39.551908	24.595607
H	34.782743	38.725626	24.290675
C	34.685276	40.859578	24.473438
H	35.401214	41.441458	23.869620
C	33.370023	40.721913	23.738334
C	34.558508	41.581610	25.819033
C	43.234560	30.854426	20.121675
C	43.252195	31.746800	21.358583
H	42.986843	31.225843	22.283527
H	44.236586	32.187474	21.563134
C	42.300619	32.936549	21.315924
O	42.128996	33.461258	22.512259
O	41.729078	33.359637	20.324488
C	40.366730	30.632557	23.125474
C	39.817882	31.231561	21.809292
H	40.610791	31.403885	21.079049
H	39.122316	30.531268	21.322035
C	39.050555	32.510905	22.001034
N	39.634099	33.700307	22.392131
H	41.089067	33.771320	22.517761
C	38.648973	34.570770	22.548679
H	38.778612	35.607651	22.829104
N	37.446914	34.022786	22.299797
C	37.690643	32.718947	21.952504
H	36.888661	32.032973	21.721159
O	35.185266	35.017832	22.628095
H	34.679068	34.640447	21.891281
H	36.406643	34.522640	22.433793

TS4

C	32.673391	37.126664	26.365997
C	31.421492	36.998434	25.602506
O	30.298084	37.005937	26.122860
N	31.531440	36.862200	24.262976
H	32.459301	36.978093	23.834404
C	30.379886	37.052759	23.389318
H	29.812569	37.898034	23.813486
C	30.850096	37.366416	22.001234
C	29.423600	35.859483	23.440290
C	36.766385	36.896122	28.757078
C	36.344413	37.401277	27.428196
O	35.172790	37.676402	27.186332
N	37.315033	37.487822	26.490711
H	38.282663	37.296326	26.754324
C	36.982641	37.750758	25.090977
H	37.929251	37.818529	24.555749
C	36.201097	36.540438	24.523593
H	36.655355	35.646299	24.963790
H	35.156989	36.577201	24.846874
O	36.243045	36.420598	23.103584
C	34.452123	36.376458	22.474327
O	33.936406	37.358377	23.032496
C	34.625893	36.434892	20.973970
C	33.714578	35.709805	20.199235

C 33.594513 35.954526 18.834614
C 34.422786 36.899187 18.229521
C 35.375911 37.583444 18.992238
C 35.457660 37.375200 20.365619
H 36.151567 37.937273 20.978700
H 36.025085 38.294037 18.496543
C 34.348544 37.176759 16.762047
O 35.302396 37.493705 16.093002
O 33.094374 37.062817 16.279244
C 32.920808 37.168464 14.849979
C 33.110694 38.559568 14.295129
H 33.628041 36.480025 14.379610
H 31.898769 36.801422 14.711621
H 32.875474 35.402964 18.240065
H 33.073474 34.973833 20.673141
C 36.391131 39.175727 25.037879
O 37.112499 40.129149 25.352736
N 35.111123 39.350526 24.690208
H 34.570539 38.569194 24.326347
C 34.566784 40.692963 24.545579
H 35.335434 41.243014 23.979878
C 33.285462 40.608921 23.746044
C 34.403333 41.416618 25.886117
C 43.368853 30.707132 19.942101
C 43.392047 31.555030 21.218289
H 43.152453 30.973216 22.122436
H 44.380595 31.983977 21.428486
C 42.427567 32.755396 21.280663
O 42.413122 33.333558 22.423575
O 41.711607 33.087607 20.322186
C 40.278382 30.517440 23.354765
C 39.753373 31.217162 22.077288
H 40.543520 31.378194 21.342937
H 38.996523 30.592644 21.580853
C 39.106398 32.564070 22.304615
N 39.844593 33.709641 22.485278
H 40.932254 33.722912 22.470983
C 39.000576 34.726967 22.661816
H 39.314495 35.749053 22.796575
N 37.727732 34.334716 22.624102
C 37.792030 32.974356 22.397148
H 36.903878 32.365103 22.332502
O 34.080105 35.157604 22.981489
H 34.504888 34.384979 22.551089
H 36.783606 35.499742 22.852693
